# Loss of the mitochondrial protein Abcb10 results in altered arginine metabolism in MEL and K562 cells and nutrient stress signaling through ATF4

**DOI:** 10.1016/j.jbc.2023.104877

**Published:** 2023-06-01

**Authors:** Marisa Miljkovic, Alexandra Seguin, Xuan Jia, James E. Cox, Jonathan Leon Catrow, Hector Bergonia, John D. Phillips, W. Zac Stephens, Diane M. Ward

**Affiliations:** 1 Division of Microbiology and Immunology, Department of Pathology, University of Utah School of Medicine, Salt Lake City, Utah, USA; 2 Department of Biochemistry, University of Utah School of Medicine, Salt Lake City, Utah, USA; 3 Metabolomics Core Research Facility, University of Utah School of Medicine, Salt Lake City, Utah, USA; 4 Iron and Heme Core Research Facility, University of Utah School of Medicine, Salt Lake City, Utah, USA; 5 Division of Hematology, Department of Medicine, University of Utah School of Medicine, Salt Lake City, Utah, USA

**Keywords:** arginine, differentiation, erythroid, metabolism, nutrient, transporter

## Abstract

Abcb10 is a mitochondrial membrane protein involved in hemoglobinization of red cells. Abcb10 topology and ATPase domain localization suggest it exports a substrate, likely biliverdin, out of mitochondria that is necessary for hemoglobinization. In this study, we generated *Abcb10* deletion cell lines in both mouse murine erythroleukemia and human erythroid precursor human myelogenous leukemia (K562) cells to better understand the consequences of Abcb10 loss. Loss of Abcb10 resulted in an inability to hemoglobinize upon differentiation in both K562 and mouse murine erythroleukemia cells with reduced heme and intermediate porphyrins and decreased levels of aminolevulinic acid synthase 2 activity. Metabolomic and transcriptional analyses revealed that Abcb10 loss gave rise to decreased cellular arginine levels, increased transcripts for cationic and neutral amino acid transporters with reduced levels of the citrulline to arginine converting enzymes argininosuccinate synthetase and argininosuccinate lyase. The reduced arginine levels in Abcb10-null cells gave rise to decreased proliferative capacity. Arginine supplementation improved both Abcb10-null proliferation and hemoglobinization upon differentiation. Abcb10-null cells showed increased phosphorylation of eukaryotic translation initiation factor 2 subunit alpha, increased expression of nutrient sensing transcription factor ATF4 and downstream targets DNA damage inducible transcript 3 (Chop), ChaC glutathione specific gamma-glutamylcyclotransferase 1 (Chac1), and arginyl-tRNA synthetase 1 (Rars). These results suggest that when the Abcb10 substrate is trapped in the mitochondria, the nutrient sensing machinery is turned on remodeling transcription to block protein synthesis necessary for proliferation and hemoglobin biosynthesis in erythroid models.

Hematopoietic lineage cells require significant metabolic remodeling during hemoglobinization (1). Mitochondria play a crucial role in sensing and signaling to make these changes. Many of these changes require transport of substrates into and out of the mitochondria, and several mitochondrial transporters have been identified based upon human diseases and model organism homolog studies. Examples include the mitochondrial serine transporter Sfxn1, which is necessary for glycine and purine synthesis (2) and the mitochondrial pyruvate carrier MCP1, which is essential for carbohydrate, fat, and amino acid metabolism (3, 4). When these transporters are altered (*e.g.*, increased or decreased expression), mitochondrial metabolism is altered resulting in signals for transcriptional changes that allow shifting from glycolysis to oxidative phosphorylation (OXPHOS) or back to glycolysis. ATP-binding cassette (Abc) transporters belong to a large superfamily of membrane proteins and are found in all kingdoms of life (5). Abc transporters hydrolyze ATP, which allows for the import and export of various substrates. Four mammalian Abcb transporters, Abcb6, Abcb7, Abcb8, and Abcb10, have been identified to localize to the mitochondria. Abcb10 (also known as ABC-mitochondrial erythroid) is expressed in most tissues including the liver, kidneys, brain, heart, and spleen but is most abundant in the fetal liver and adult bone marrow, the primary sites for terminal hematopoiesis (6). There has been some debate over what substrate is exported by Abcb10, but a recent study suggests that Abcb10 transports a breakdown product of heme, biliverdin (7).

Abcb10 knockout mice shows severe anemia at day 10.5 of development, and it is embryonically lethal by day 12.5 of gestation (8). Abcb10 has also been demonstrated to enhance heme biosynthesis by forming a complex with mitoferrin 1 (Mfrn1) and ferrochelatase (Fech) (9, 10). Murine erythroleukemia (MEL) cells stably expressing shRNA directed against *Abcb10* showed that reductions in Abcb10 affected heme synthesis but no accumulation of protoporphyrin IX (PPIX) suggesting that iron was not limiting and Fech was functional (11). In same study, we also demonstrated that reductions in Abcb10 resulted in decreased expression of heme biosynthesis genes *aminolevulinic acid synthase 2 (Alas2*) and *Fech* and mitochondrial iron acquisition gene *Mfrn1* and *β-globin* transcripts. This suggests that the substrate exported by Abcb10 provides a signal for the induction of transcription of hemoglobin biosynthesis genes upon differentiation.

We utilized both metabolic and transcriptional analysis to identify how loss of Abcb10 affects the transcriptional program of heme biosynthesis. To accomplish this, we deleted Abcb10 in two different hematopoietic cell lines. We have determined the loss of Abcb10 affects arginine metabolism in MEL and K562 cell models of erythroid differentiation. Decreased arginine levels induced the increased expression of nutrient sensing transcription factor ATF4 and its downstream targets CHOP and CHAC1 that limit protein synthesis until the nutrients stress response is satisfied, and the necessary metabolic shifts permit hemoglobinization.

## Results

### Loss of Abcb10 results in diminished hemoglobinization in differentiated MEL and K562 cells

**Figure 1:**
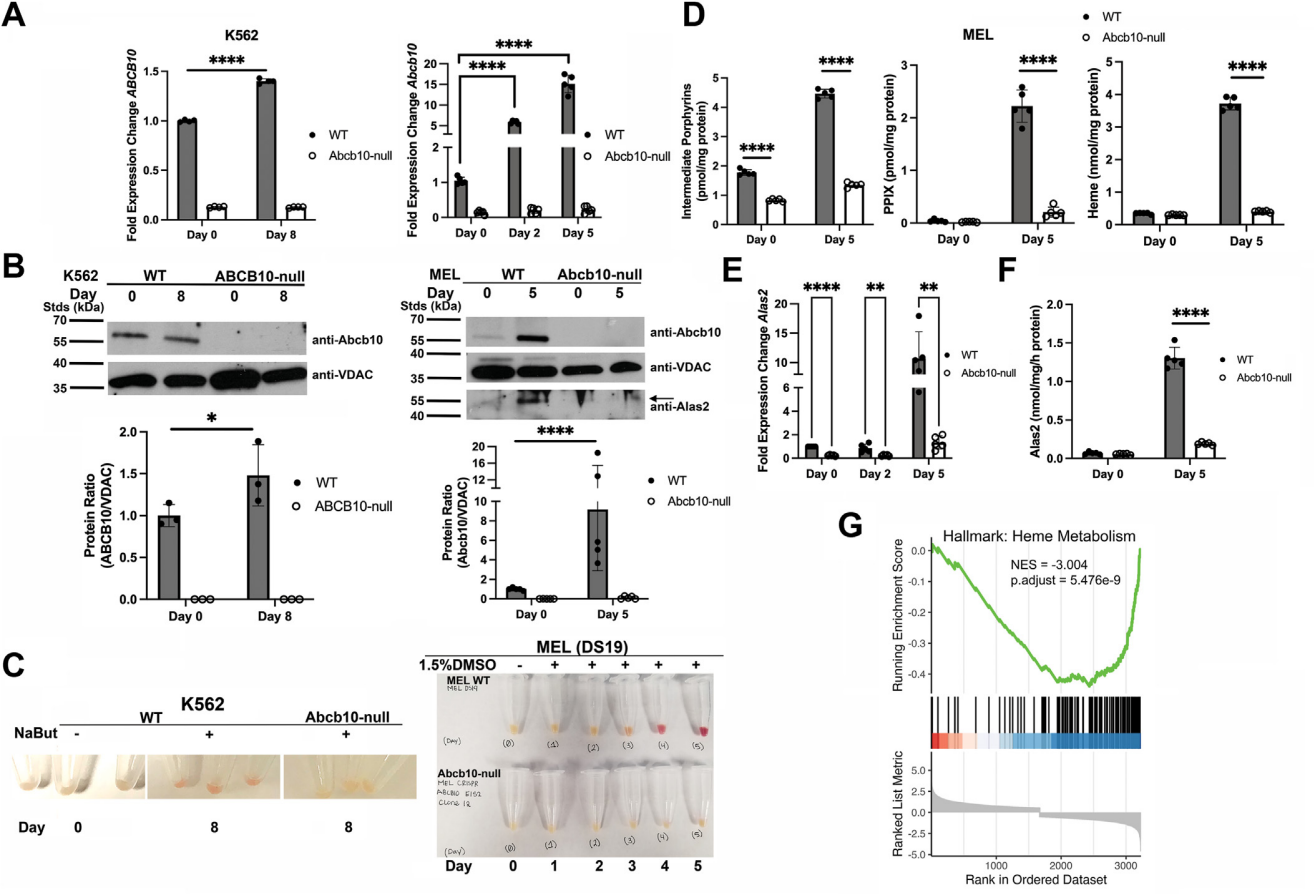
**Loss of Abcb10 using CRISPR/Cas9 strategy resulted in reduced expression of heme synthesis enzymes with corresponding reductions in hemoglobinization.** WT K562 and MEL cells were transduced with control (mCherry) or *ABCB10/Abcb10*-targeted CRISPR/Cas9 lentiviral constructs as described in Experimental procedures. *A*, RT-qPCR was performed on isolated clones that were differentiated for either 8 days (K562) or 2 and 5 days (MEL) as described in Experimental procedures using actin transcripts as a control. Bar graphs show the mean ± SD, n = 3 to 5 experiments with Student’s *t* test significance *p* ≤ 0.05. *B*, Western blot analysis for Abcb10 protein was performed on isolated mitochondria from cells as in (*A*) using VDAC (porin) as a loading control. A representative blot is shown, and experimental replicate blots were quantified using Fiji ImageJ software. Bar graphs show the mean ± SD, n = 3 to 5 experiments with Student’s *t* test significance *p* ≤ 0.05. For MEL cells, Alas2 protein levels were also determined by Western blot. *C*, a time course of hemoglobinization of cells as in (*A*) is shown for WT K562, Abcb10-null K562 cells (*left panels*, three replicates), and WT MEL and Abcb10-null MEL cells (*right panel*). *Red color* observed in the cell pellets indicates hemoglobinization. *D*, porphyrin intermediates, PPIX, and heme levels of WT and Abcb10 null differentiated MEL cells were determined as described in Experimental procedures (n = 5 replicates/experiment). A representative experiment is shown. Bar graphs show the mean ± SD, n = 3–5 experiments) with Student’s *t* test significance *p* ≤ 0.05. *E*, RT-qPCR transcripts for *Alas2* were determined in differentiated WT and Abcb10-null MEL cells. Bar graphs show the mean ± SD, n = 3 to 5 experiments with Student’s *t* test significance *p* ≤ 0.05. *F*, Alas2 activity in cells as in (*E*) were determined as described in Experimental procedures (n ≤ 3 biologic replicates with a minimum of three experimental replicates). Bar graphs show the mean ± SD, with Student’s *t* test significance *p* ≤ 0.05. *G*, RNA Seq was performed on undifferentiated and differentiated WT and Abcb10-null MEL cells (n = 5 replicates) as described in Experimental procedures. Gene Set Enrichment Analysis on Hallmark gene sets showed marked reductions in heme metabolism at Day 5 of differentiation. MEL, murine erythroleukemia; PPIX, protoporphyrin IX.

Loss of Abcb10 is embryonic lethal, and loss of Abcb10 in hematopoietic cells results in severe anemia (8). We have previously shown that reductions in Abcb10 results in partial hemoglobinization in the MEL model for hemoglobinization (11). The partial hemoglobinization was thought to be due to residual Abcb10 protein. To determine if there is a complete loss of hemoglobinization in the absence of Abcb10, K562 and MEL cells were transduced with lentiviral CRISPR/Cas9 constructs deleting specific regions of exons of either the human (K562) or murine (Mel) *ABC10* genes (Table 1). Cells were sorted for positive transduction by mCherry, BFP, or GFP, and single cell clones were analyzed by RT-qPCR and Western blot. We identified both K562 and MEL cell CRISPR/Cas9 clones that showed significant reductions in *ABCB10* or *Abcb10* transcripts in both undifferentiated and differentiated cells compared to wildtype (WT) K562 or MEL cells (Fig. 1*A*). The loss of h*ABCB10*/m*Abcb10* transcripts led to loss of Abcb10 protein in both K562 and MEL cells (Fig. 1*B*). We note that Abcb10 induction during differentiation was much more dramatic in MEL cells compared to K562. In K562 cells, we observed that Abcb10 protein was present even in undifferentiated cells suggesting constitutive expression. To determine if there were differences in hemoglobinization, WT K562 and MEL cells along with Abcb10-null cells were differentiated using either Na-butyrate (K562) for 8 days or 1.5% dimethyl sulfoxide (DMSO)(MEL) for 5 days. Both Abcb10-null K562 and MEL cells showed a loss of hemoglobinization compared to WT (Fig. 1*C*). We observed some Abcb10 CRIPSR-transduced MEL clones that showed only partial reductions in hemoglobinization, which corresponded with remaining Abcb10 protein (Fig. S1*A*, clone J). We focused on clones that showed a complete loss of Abcb10 protein (K562 clone #40 and MEL clone #12), referred to as Abcb10 null or Abcb10^−/−^. We utilized the MEL cell model to quantify changes in heme and intermediate porphyrins, as hemoglobinization was more prominent in MEL cells. Abcb10-null MEL cells showed significantly reduced intermediate porphyrins, PPIX, and heme levels compared to WT MEL cells (Fig. 1*D*).

**Table 1:** CRISPR/Cas9 target sequences

Species	Target gene	Target sequence
Human	*ABCB10*	E1S1 GTCGCACGCAGCGCCATGCG
Mouse	*Abcb10*	E1S2 CGTACGCGCGTGGACCTC
Mouse	*Abcb10*	E3S9 TCGGATACCGCACTCC

E = exon; S = site.

There are two rate-limiting enzymes in heme synthesis: Alas2, which makes ALA from glycine and succinyl CoA, and Fech, which inserts iron into the PPIX ring (12). *Alas2* is known to be induced during erythroid differentiation. Previously, we showed that there was decreased *Alas2* expression when Abcb10 levels were reduced using siRNA (11). Alas2 levels were also downregulated by the complete loss of Abcb10. *Alas2* transcripts were reduced in Abcb10-null cells (Fig. 1*E*), and this reduction in *Alas2* expression corresponded with decreased Alas2 protein (Fig. 1*B*) and decreased Alas2 activity in the absence of Abcb10 (Fig. 1*F*). We performed RNA seq on MEL cells during differentiation to identify other pathways that might be affected by loss of Abcb10. Gene set enrichment analysis (GSEA) revealed that transcripts for other enzymes in the heme biosynthesis pathway that are induced during differentiation were decreased in the absence of Abcb10, especially on Day 5 where transcripts for hemoglobinization are highly induced in WT MEL cells (Fig. 1*G*). Transcripts for *Alad*, *Urod, Cpox, Ppox*, and *Fech* were reduced in the absence of Abcb10, whereas *UroS* transcripts were induced to similar levels to WT MEL cells (Fig. S1*B*). Together, the loss of Abcb10 decreased the induction of hemoglobinization and therefore intermediate porphyrins and heme did not accumulate compared to WT cells.

### Loss of Abcb10 causes cellular arginine depletion and citrulline and ornithine accumulation

**Figure 2:**
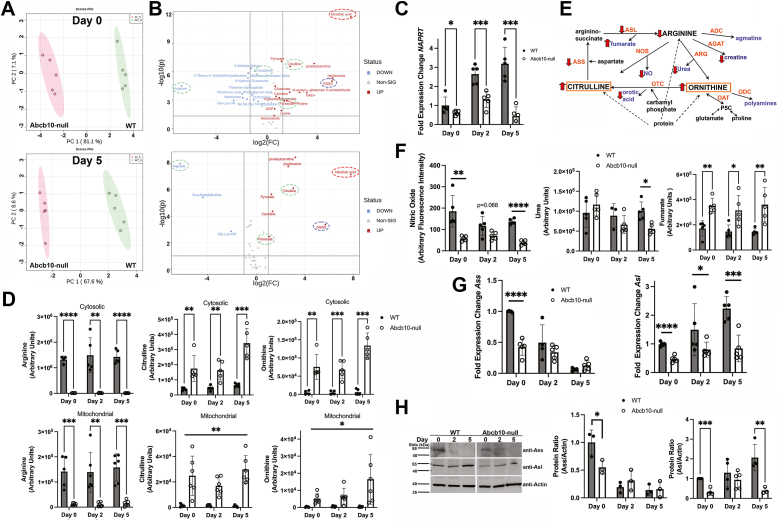
**Targeted metabolomics revealed changes in arginine metabolism in the absence of Abcb10.***A*, metabolomic analysis was performed on undifferentiated and differentiated WT and Abcb10-null MEL (n = 5 replicates) as described in Experimental procedures. Principal component analysis (PCA) was performed on metabolomic results from Day 0 and Day 5 of differentiation. A representative experiment is shown. *B*, volcano plots of changes in metabolites observed in Abcb10-null MEL cells at Day 0 and Day 5 are shown. Significance is shown at greater than 1.5-fold change (log2 scale) (n = 2 experimental replicates). *Hash circled* in *green* are components of the arginine metabolism pathway, in *red* nicotinic acid and in *blue* GSSG. *C*, transcript levels for *NAPRT* with *actin* as a loading control was performed using RT-qPCR as described in Experimental procedures (n = 2 technical replicates and five biologic replicates). Bar graphs show the mean ± SD, with Student’s *t* test significance *p* ≤ 0.05. *D*, targeted metabolomics for arginine, citrulline, and ornithine were determined from cells treated as in (*A*) as described in Experimental procedures. The data are expressed as arbitrary units (each experiment n = 5–6 replicates; n = 2 biologic replicates). Bar graphs show the mean ± SD, with Student’s *t* test significance *p* ≤ 0.05. *E*, arginine metabolism pathway—citrulline is a metabolite of arginine metabolism that is generated either from the breakdown of modified arginine (citrulline) in proteins, the activity of nitric oxide synthetase (NOS) generating NO or through arginase (ARG) that is responsible for converting toxic NH_4_^+^ to nontoxic urea resulting in the conversion of arginine to ornithine, which can then be combined with carbamoyl phosphate by the enzymatic reaction of ornithine transcarbamylase (OTC) to generate citrulline and the metabolite orotic acid. Citrulline can then be reconverted to arginine by the enzymes argininosuccinate synthase (ASS) and argininosuccinate lyase (ASL) with the byproduct fumarate. Ornithine is produced by three pathways: (1) breakdown of modified arginine (ornithine) in proteins, (2) breakdown of arginine by arginase in the urea cycle, or (3) synthesized *de novo* from glutamate and proline *via* ornitine aminotransferase (OAT). Reductions in arginine can also result from breakdown by arginine decarboxylase (ADC) generating agmatine or breakdown by arginine:glycine amidinotransferase (AGAT) generating creatine. *Red arrows* denote changes observed in the absence of Abcb10 as measured by metabolomics (*A*, *B*, and *D*) and Western blot (*H*). *F*, targeted measurements of metabolites including NO as describe in Experimental procedures, urea and fumarate were determined from cells as in (*D*). Bar graphs show the mean ± SD, with Student’s *t* test significance *p* ≤ 0.05. *G*, transcript levels for *Ass* and *Asl* with *actin* as a loading control were performed using RT-qPCR as described in Experimental procedures (n = 2 technical replicates and five biologic replicates). Bar graphs show the mean ± SD, with Student’s *t* test significance *p* ≤ 0.05. *H*, Western blot analysis for Ass, Asl, and actin were performed on cells as in (*A*) (n = 3 biologic replicates). Blots were quantified using Fiji Image J as described in Experimental procedures using actin as a loading control. Bar graphs show the mean ± SD, with Student’s *t* test significance *p* ≤ 0.05. MEL, murine erythroleukemia.

To determine other cellular consequences of Abcb10 loss, we performed metabolomics on mitochondria and cytosol from differentiated WT and Abcb10-null MEL cells. We reasoned that the substrate transported by Abcb10 might be “trapped” inside the mitochondria, thus altering mitochondrial and cytosolic metabolites. Differences in metabolites in the absence of Abcb10 were detected at each day of differentiation. Principle component analysis grouped cells according to genotype confirming that metabolites were consistently altered in the absence of Abcb10 (Fig. 2*A*). Volcano plots showed the most prominent changes were in arginine metabolism, oxidized glutathione GSSG, and nicotinic acid (NA) levels (Fig. 2*B*). Oxidized glutathione (GSSG) was dramatically increased in the absence of Abcb10 suggestive of increased oxidative stress; however, we did not observe any changes in reduced glutathione (GSH) levels (Fig. S2*A*). We observed large increases in NA in Abcb10-null MEL cells (Fig. 2*B*). NA can be used in the salvage pathway to generate nicotinamide adenine dinucleotide (NAD) through the activity of the enzyme NA phosphoribosyltransferase (NAPRT) (13). We observed increased NAD at Day 0, but no increase in NAD was observed at Day 5. RT-qPCR revealed that *NAPRT* transcripts were increased during MEL cell differentiation in WT cells, but transcripts were not consistently increased in the absence of Abcb10 (Fig. 2*C*). These results provide an explanation for the accumulation of NA, but not for the decrease in *NAPRT* transcripts. We also observed significant increases in cytosolic and mitochondrial pyruvate (Fig. S2*B*), suggesting that glycolysis is increased, and/or the Krebs cycle utilization of pyruvate is reduced.

Targeted LC-MS/MS metabolomics showed the most dramatic changes in arginine, citrulline, and ornithine with significant diminutions in arginine and accumulation of citrulline and ornithine in both the mitochondria and cytosol in the absence of Abcb10 in MEL cells (Fig. 2*D*). An abbreviated arginine metabolism pathway is shown in Fig. 2*E*. To identify the mechanism of citrulline and ornithine accumulation in the absence of Abcb10, we first measured nitric oxide and urea levels. Nitric oxide levels were decreased in the absence of Abcb10, whereas urea levels were only significantly reduced on day 5 of differentiation (Fig. 2*F*). Importantly, nitric oxide has been shown to inhibit hemoglobinization in MEL cells (14), but we observed reduced levels of nitric oxide in the absence of Abcb10, suggesting that the loss of hemoglobinization was not due to elevated nitric oxide. To determine if citrulline accumulation in the absence of Abcb10 was a consequence of ornithine and carbomoyl phosphate being converted to citrulline by the enzyme ornithine transcarbamylase, we measured the metabolite orotic acid. We saw a slight reduction in cytosolic orotic acid at day 5 of differentiation, but no changes in mitochondrial orotic acid were observed in Abcb10-null cells (Fig. S2*C*). Similarly, there was a trend toward decreased levels of creatine, a breakdown product of arginine, supporting that arginine reductions were not due to increased breakdown in the absence of Abcb10. Two amino acids that can be utilized to make ornithine are proline and glutamate. Proline levels were either equivalent or elevated in the absence of Abcb10, although we noted that proline levels were quite variable, while glutamate levels trended downward in the absence of Abcb10 (Fig. S2*D*). As ornithine also accumulated, this suggests that there is not a defect in ornithine nor citrulline synthesis and that the excess of these two molecules may be due to a defect in regeneration of arginine.

Arginine is converted to ornithine and urea through the activity of the enzyme arginase. Ornithine levels were elevated in the absence of Abcb10, but there was no increase in cellular urea (Fig. 2*F*). If anything, we observed a trend toward decreased cellular urea levels in Abcb10-null MEL cells. This may reflect decreased cellular arginine or increased urea released into the growth media. We were unable to detect urea in the growth media of either WT or Abcb10-null cells. Elevated citrulline and ornithine could be a consequence of arginine modification on associated proteins. We were unable to detect any citrullination of proteins in MEL cells (data not shown); however, we observed a slight increase in peptidyl arginine deiminase 3 (*Padi3*) transcripts, the enzyme responsible for modification of arginine to citrulline/ornithine, in Abcb10-null cells at days 0 and 5 compared to WT (Fig. S2*E*). Transcripts for other *Padi* genes *Padi1, 2,* and *4* were not detected in the RNA seq results. The fact that there is increased oxidized glutathione (GSSG) even at day 0 in the absence of Abcb10 supports that there is increased oxidative stress, which may be due to increased citrullinated proteins.

Previous studies have suggested that Abcb10 is important in regulating oxidative stress, although the observation that citrulline levels would change in the absence of Abcb10 has not been previously appreciated (7, 15, 16). Citrulline is converted back to arginine by the action of two enzymes argininosuccinate synthetase (Ass) and argininosuccinate lyase (Asl). Transcripts levels for *Ass* decreased during WT differentiation and Abcb10-null cells showed reduced expression, most significantly at day 0, while *Asl* transcripts were increased during differentiation in WT cells but were consistently lower in the absence of Abcb10 (Fig. 2*G*). Ass protein levels trended downward in WT cells during differentiation agreeing with transcript levels, and loss of Abcb10 showed reduced Ass most significantly at Day 0 compared to WT cells (Fig. 2*H*). Asl protein levels trended upward in WT cells over differentiation agreeing with transcripts and Asl protein levels were decreased in the absence of Abcb10 compared to WT cells. These results suggest that Ass and Asl levels may be insufficient for regenerating arginine in the absence of Abcb10.

### Arginine supplementation improves hemoglobinization in the absence of Abcb10

**Figure 3:**
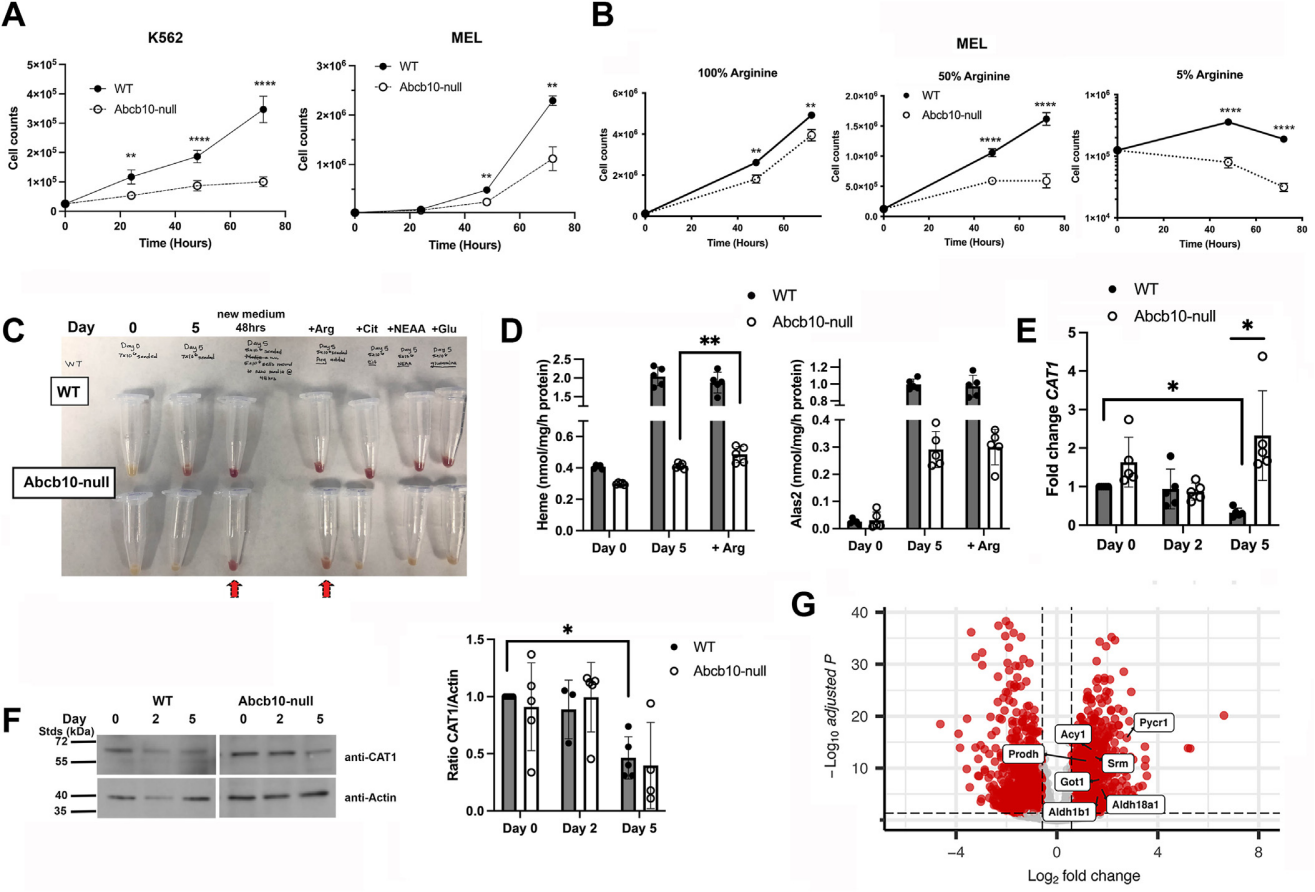
**Loss of Abcb10 results in arginine-dependent proliferation and hemoglobinization.***A*, WT and Abcb10-null K562 and MEL cells were grown for 0 to 72 h and cell proliferation measured. Graphs show the mean ± SD, n = 5 biologic replicates, with two-way ANOVA, significance *p* ≤ 0.05. *B*, WT and Abcb10-null MEL cells were grown in 100%, 50%, or 5% arginine-containing medium for 0 to 72 h and proliferation measured. Graphs show the mean ± SD, n = 5 biologic replicates, with two-way ANOVA, significance *p* ≤ 0.05. *C*, MEL cells as in (*B*) were differentiated for 0 or 5 days in normal growth medium ± 2X arginine (Arg), citrulline (Cit), nonessential amino acids (NEAA), glutamine (Glu), or growth medium replace at day 3 of differentiation. *D*, heme levels and Alas2 activity in WT and Abcb10-null MEL cells as in C ± Arg supplementation at Day 0 and Day 5 were measured as described in Experimental procedures. Bar graphs show the mean ± SD, n = 5 biologic replicates, with Student’s *t* test significance *p* ≤ 0.05. *E*, *CAT1* transcripts were determined in undifferentiated and differentiated WT and Abcb10-null MEL cells using RT-qPCR. Transcripts are expressed as fold change relative to *actin* levels of WT cells Day 0 as described in Experimental procedures. Bar graphs show the mean ± SD, n = 5 biologic replicates, with Student’s *t* test significance *p* ≤ 0.05. *F*, Western blot for CAT1 and actin levels from undifferentiated and differentiated WT and Abcb10-null MEL cells were determined as described in Experimental procedures. Quantification from 4 to 5 biologic replicate experiments was done using Fiji ImageJ software. Bar graphs show the mean ± SD, with Student’s *t* test significance *p* ≤ 0.05. *G*, volcano plot from RNA seq comparing WT and Abcb10-null MEL at Day 5 with core enriched genes labeled from the KEGG arginine and proline metabolism gene set that were significantly enriched (NES = 2.197, *p* value = 0.0192). *Dotted lines* indicate cutoffs used for GSEA analysis. Eight data points with very high -Log10 adjusted *p* values are above the y-axis range and are not shown. GSEA, Gene set enrichment analysis; KEGG, Kyoto Encyclopedia of Genes and Genomes; MEL, murine erythroleukemia.

Decreased media arginine has been shown to limit proliferation and hemoglobinization in human erythroleukemia cell line K562 and primary human erythroid precursor CD34+ cells suggesting that maintaining arginine levels is important in erythroid differentiation (17, 18, 19, 20). We noted that the absence of Abcb10 reduced cell proliferation in both K562 and MEL cells (Fig. 3*A*). As Abcb10-null K562 cells had dramatically reduced proliferative capacity, we utilized MEL cells to measure cell proliferation upon arginine limitation. We observed that arginine limitation reduced both WT and Abcb10-null MEL cell proliferation and that loss of Abcb10 resulted in even greater decreased proliferation compared to WT cells (Fig. 3*B*). We noted that addition of new media to MEL cells induced some hemoglobinization in the absence of Abcb10. Upon further exploration, we found that addition of more arginine was sufficient to induce hemoglobinization, whereas addition of citrulline, nonessential amino acids, or glutamine did not improve hemoglobinization in Abcb10-null cells (Fig. 3*C*). Importantly, these additions did not negatively affect WT cell hemoglobinization. We note that hemoglobinization in arginine supplemented Abcb10-null cells was not equivalent to levels seen in WT MEL cells suggesting that there may be other changes induced by the loss of Abcb10 that downregulate hemoglobinization. Concomitant with increased “redness” in Abcb10-null cells grown in 2X arginine, we observed a slight increase in heme levels but no change in Alas2 activity, while heme and Alas2 activity were not affected in WT cells (Fig. 3*D*).

Arginine uptake in red cell precursors is predominantly mediated by the plasma membrane cationic amino acid transporter 1 (CAT1), and the loss of CAT1 in developing erythroid precursors prevents hemoglobinization (20). To determine if there were changes in CAT1 levels, we measured transcripts and protein. *CAT1* transcripts were reduced over time of differentiation in WT MEL cells, whereas in response to Abcb10 loss, *CAT1* transcripts remained high (Fig. 3*E*). Western blot analysis showed no difference in CAT1 protein levels Abcb10-null MEL cells compared to WT levels with a trend toward decreased CAT1 protein levels (Fig. 3*F*). It is also possible that uptake of arginine is altered in the absence of Abcb10. To examine this possibility, we measured ^3^H-arginine uptake in WT and Abcb10-null cells. Abcb10-null cells showed a slight increase in ^3^H-arginine uptake compared to WT cells (Fig. S3*A*) supporting that arginine import is not defective. RNA Seq revealed that several transcripts for arginine and proline metabolism were upregulated in the absence of Abcb10 during differentiation (Fig. 3*G*). RNA Seq also revealed that the loss of Abcb10 in MEL cells resulted in increased transcripts for several other amino acid transporters of the solute carrier family 7 (Slc7), including *Slc7a5, Slc7a6, Slc7a7,* and *Slc7a11* during differentiation compared to WT cells (Fig. S3*B*). *Slc7a11* transcripts were the most dramatically increased in the absence of Abcb10. Unfortunately, we were unable to detect Slc7a11 protein in MEL or K562 cells using Western blot analysis (data not shown). Overall, our results support that amino acid changes are sensed by the Abcb10-null cells, but the imported arginine may be insufficient or that limited arginine regeneration due to a repression in either transcription and/or translation gives rise to an inability of erythroid precursors to differentiate.

Abcb10 is proposed to be a mitochondrial biliverdin exporter and that exported biliverdin can be converted in the cytosol to bilirubin to act as an antioxidant (21). Unfortunately, our metabolomics analysis was unable to detect heme and heme breakdown products. Regulating the cytosolic levels of biliverdin/bilirubin by requiring a mitochondrial exporter provides another layer of antioxidant control. One prediction of Abcb10 as a biliverdin transporter would be that providing exogenous bilirubin to differentiating MEL cells would improve hemoglobinization. To determine if changes in biliverdin/bilirubin levels affected MEL cell hemoglobinization, we grew cells in the presence of 10 μM bilirubin, which is more lipophilic than biliverdin and is able to cross membranes. Addition of bilirubin did not improve hemoglobinization of Abcb10-null MEL cells and, in fact, reduced hemoglobinization in even WT MEL cells (Fig. S3*C*). To our knowledge, this is the first report that excess bilirubin reduces heme levels in erythroid cell culture models. Bilirubin is a potent antioxidant, and diminished levels of cytosolic bilirubin, possibly due to trapping of biliverdin in the mitochondria, would be predicted to cause increased reactive oxygen species. Our metabolomics data confirmed increased oxidative stress in Abcb10-null cells as there was increased oxidized glutathione (GSSG) (Fig. 2*B*). This suggests that Abcb10, as a mitochondrial biliverdin exporter, contributes to cellular antioxidant levels. Our results support that there is a connection between arginine metabolism and Abcb10 biliverdin export for erythroid differentiation.

### The absence of Abcb10 affects mitochondrial respiration

**Figure 4:**
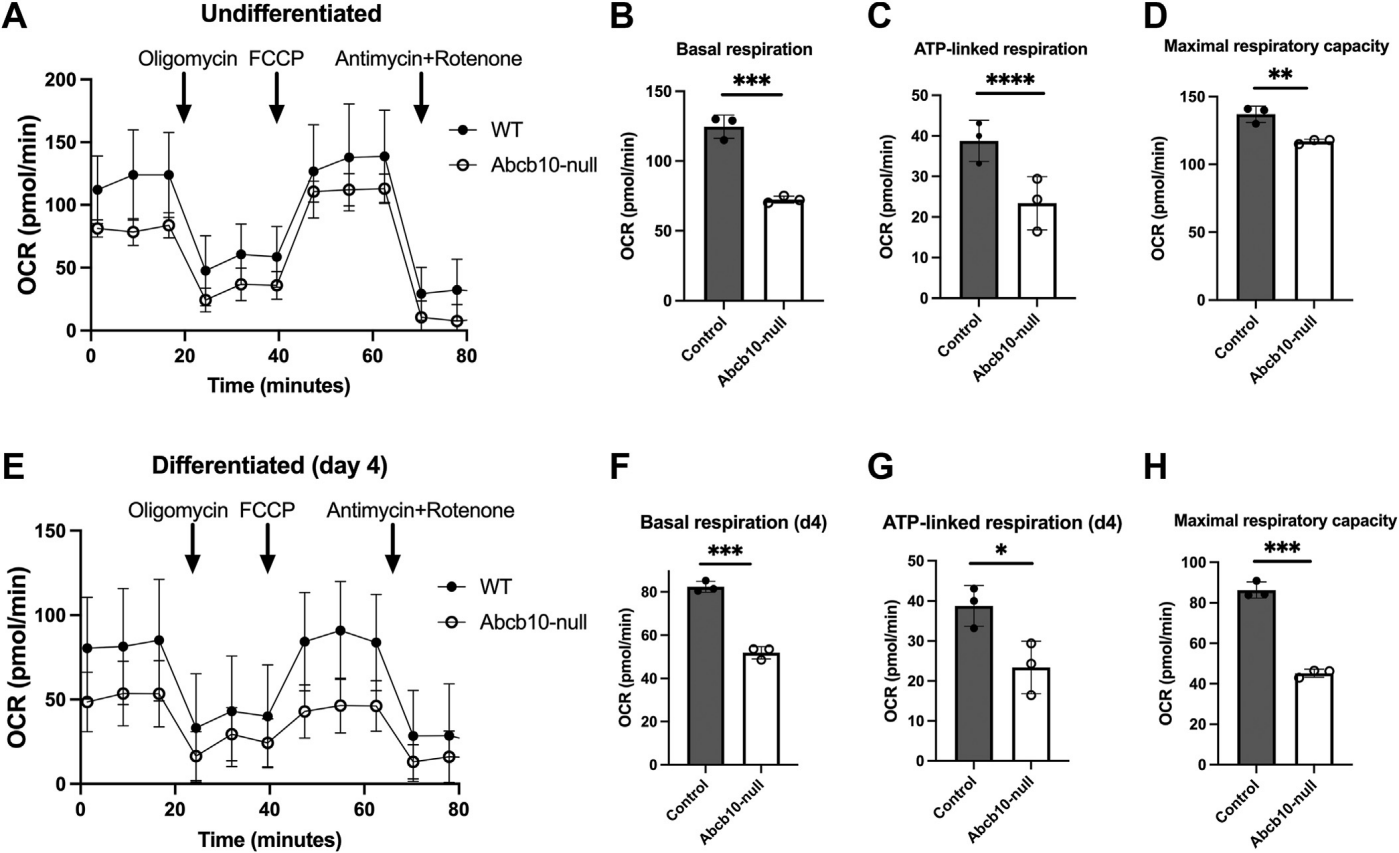
**Loss of Abcb10 results in diminished mitochondrial respiration.***A*, Seahorse analysis was performed on undifferentiated WT and Abcb10-null MEL cells as described in Experimental procedures. A representative experiment is shown. *B–D*, (*B*) basal respiration, (*C*) ATP-linked respiration, and (*D*) maximal respiratory capacity from cells as in (*A*) were determined. (n = 5 technical replicates and three biological replicate experiments). Bar graphs show the mean ± SD with Student’s *t* test significance *p* ≤ 0.05. *E*, Seahorse analysis was performed on differentiated WT and Abcb10-null MEL cells. A representative experiment is shown. *F–H*, (*F*) basal respiration, (*G*) ATP-linked respiration, and (*H*) maximal respiratory capacity determined (n = 3 biologic replicate experiments). (n = 5 technical replicates and three biological replicate experiments.). Bar graphs show the mean ± SD with Student’s *t* test significance *p* ≤ 0.05. MEL, murine erythroleukemia.

Previous studies of Abcb10 using cell culture models of erythroid differentiation have not determined if mitochondrial functions beyond heme synthesis are affected in the absence of Abcb10. Alterations in arginine metabolism are known to affect mitochondrial function. A previous study determined that arginine metabolism was altered in cancer cells (22) (see (23, 24) for review). The mechanism was recently identified as mitochondrial stress signaling transcriptional reprogramming that resulted in aspartate exhaustion (25). Supplementation of aspartate or loss of mitochondria protected cancer cells from arginine depletion–mediated cell death. Aspartate supplementation, however, did not improve hemoglobinization of Abcb10-null MEL cells (Fig. S4*A*). That arginine levels were dramatically reduced in Abcb10-null cells suggests that there is an energy metabolism consequence due to Abcb10 loss. Several studies report that during differentiation erythroid precursors shift energy metabolism from glycolysis to OXPHOS prior to the loss of mitochondria (26, 27). Mitochondrial OXPHOS, measured as the oxygen consumption rate, was reduced in the absence of Abcb10 in undifferentiated and differentiated MEL cells (Fig. 4, *A* and *E*). The Seahorse analysis also revealed reduced basal respiration, reduced ATP-linked respiration, and reduced maximum respiratory capacity in undifferentiated Abcb10-null cells (Fig. 4, *B*–*D*) as well as differentiated cells compared to WT MEL cells (Fig. 4, *F*–*H*). We also measured extracellular acidification rate as a measure of glycolysis. We noted reduced levels of glycolysis in the absence of Abcb10 at Day 0 of differentiation as well as Day 4 (Fig. S4*B*). We did not, however, observe increased mitochondrial oxidants in the absence of Abcb10 as assessed by MitoSOX levels (Fig. S4*C*). Together the Seahorse analysis suggests that loss of Abcb10 affects both OXPHOS and glycolysis.

### Abcb10 loss in MEL cells induces a nutritional stress response through ATF4

**Figure 5:**
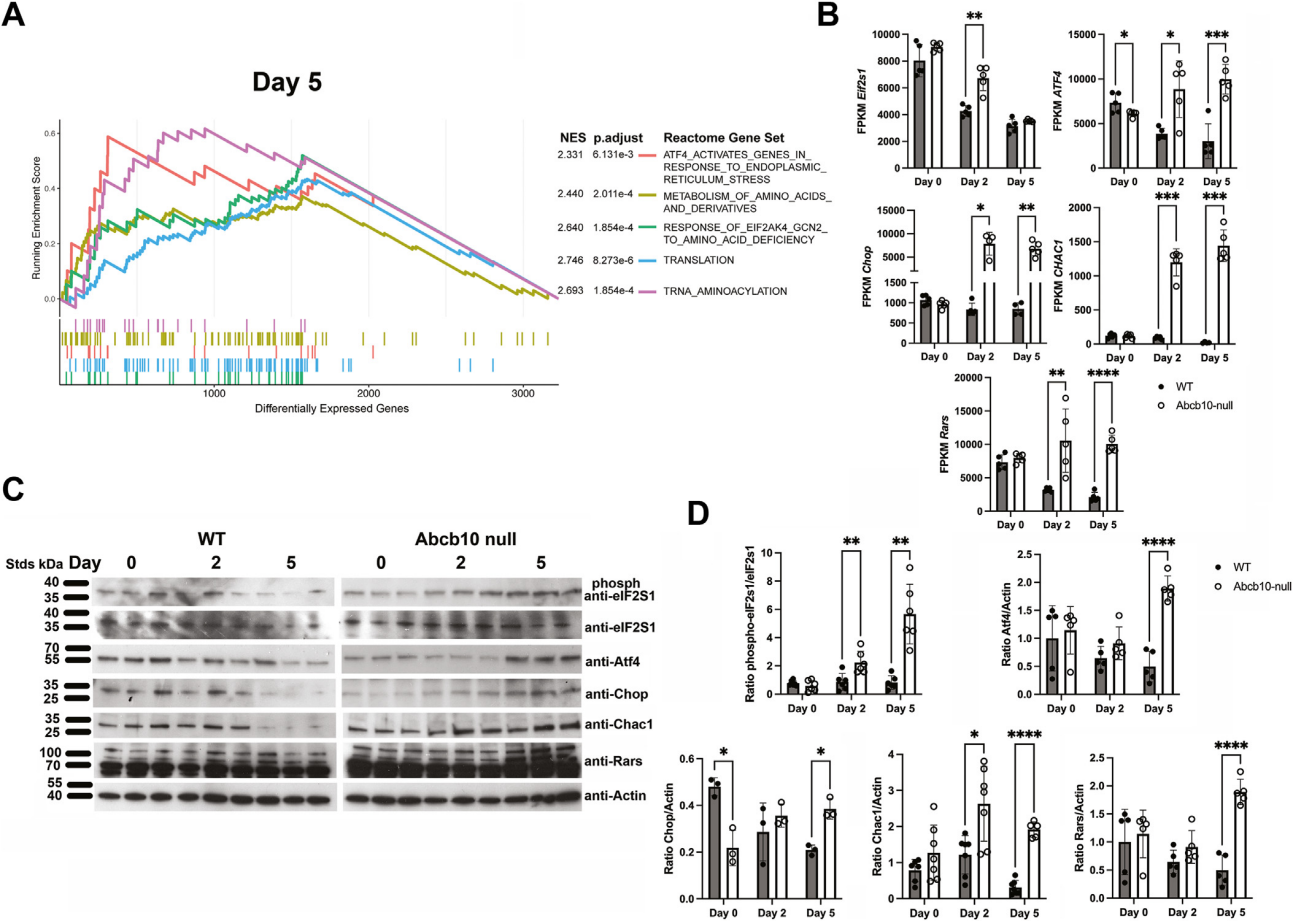
**Loss of Abcb10 results in stress signaling through eIF2S1 phosphorylation and upregulation of *ATF4* and downstream nutritional stress sensors *CHOP*, *CHAC1* and *RARS*.***A*, RNA Seq from WT and Abcb10-null MEL cells during differentiation was performed (n = 5 biologic replicates). GSEA on reactome gene sets shows enrichment of pathways involved in stress response and amino acid metabolism at day 5. *B*, transcripts for *eIF2S1*, *ATF4*, downstream target of ATF4, including *Chop*, *CHAC1*, and *Rars* in WT and Abcb10-null MEL cells at Day 0, 2, and 5 of differentiation from (*A*) were expressed as FPKM. Bar graphs show the mean ± SD, n = 3–5 experiments with Student’s *t* test significance *p* ≤ 0.05. *C*, Western blot analysis for eIF2S1, phospho-eIF2S1, Atf4, Chop, Chac1, Rars, and actin in WT and Abcb10-null MEL cells at Day 0, 2, and 5 of differentiation was performed as described in Experimental procedures. *D*, blots were quantified using Fiji Image J as described in Experimental procedures using actin as a loading control. (n = 2–3 biologic replicate/experiment; n = 3–6 experiments). The ratios of phosphorylated eIF2S1/total eIF2S1 and protein/actin are shown. Bar graphs show the mean ± SD with Student’s *t* test significance *p* ≤ 0.05. eIF2S1, eukaryotic initiation factor-2S1; GSEA, Gene set enrichment analysis; MEL, murine erythroleukemia.

Alterations in arginine metabolism are known to affect transcriptional remodeling. A precedent for this exists in T cells where intracellular arginine level alterations were found to result in global metabolic changes including the ability to shift between glycolysis and OXPHOS, which requires transcriptional remodeling. The sensors of this remodeling were identified as transcriptional regulators that responded to cellular arginine levels (28). In those studies, arginine as well as citrulline and ornithine were reduced during T cell activation. Here, we report that Abcb10 loss dramatically reduced arginine levels but elevated citrulline and ornithine levels. To get a global transcriptional view of the consequences of Abcb10 loss, we performed RNA Seq on WT and Abcb10-null MEL cells at day 0, 2, and 5 of differentiation. We noted that transcripts for genes involved in heme synthesis were reduced in Abcb10-null MEL cells and confirmed by RT-qPCR (Fig. 1, *E* and *G*). During cellular stresses such as amino acid (arginine) limitation, the eukaryotic initiation factor-2S1 (eIF2S1) is phosphorylated leading to altered gene expression designed to alleviate the cellular stress (29, 30). GSEA of RNA Seq results revealed changes in stress response genes in the nutrient sensing pathway of transcriptional activating transcription factor ATF4 as well as metabolism of amino acids, response of EIF2AK4 to amino acid deficiency, translation, and tRNA aminoacylation in the absence of Abcb10 upon differentiation with the most dramatic changes seen at day 5 of differentiation (Fig. 5*A*). ATF4 is a key component of this amino acid starvation response (31, 32). Elevated ATF4 levels are known to induce the expression of a cascade of transcriptional regulators including Chop (a CCAAT/enhancer binding protein), CHAC1 (a gamma-glutamylcyclotransferase), and Rars (arginyl-tRNA synthetase 1) that function to reduce cellular stress (29, 32, 33). Rars catalyzes the aminoacylation of tRNA by their cognate amino acid (34). During MEL cell differentiation, *ATF4* expression was decreased in WT cells, whereas, *ATF4* transcripts remained high in the absence of Abcb10 (Fig. 5*B*). Concomitant with those changes, we saw increased expression of *Chop, CHAC1,* and *Rars* in Abcb10-null MEL cells during differentiation. These observation predict increased phosphorylation of EIF2S1alpha in the absence of Abcb10. Indeed, we observed increased phosphorylation of EIF2S1alpha in the absence of Abcb10 during differentiation (Fig. 5*C*). Further, this elevation in nutrient stress transcripts equated to elevated ATF4 protein levels in differentiating Abcb10-null cell compared to WT MEL cells (Fig. 5*D*). CHAC1, Chop, and Rars protein levels showed a similar trend with increased protein levels in differentiating Abcb10-nulls cells, while those same proteins showed reduced levels in WT cells, which corresponded with decreased transcripts during differentiation. Together, these results suggest that the loss of Abcb10 gives rise to a stress response that alters gene expression and coordinates translational repression until further nutrient deprivation is fixed. This translational repression mechanism provides further explanation for why loss of the Abcb10 transport blocks hemoglobinization in red cell models and identifies Abcb10 transport as important in cellular metabolism.

## Discussion

**Figure 6:**
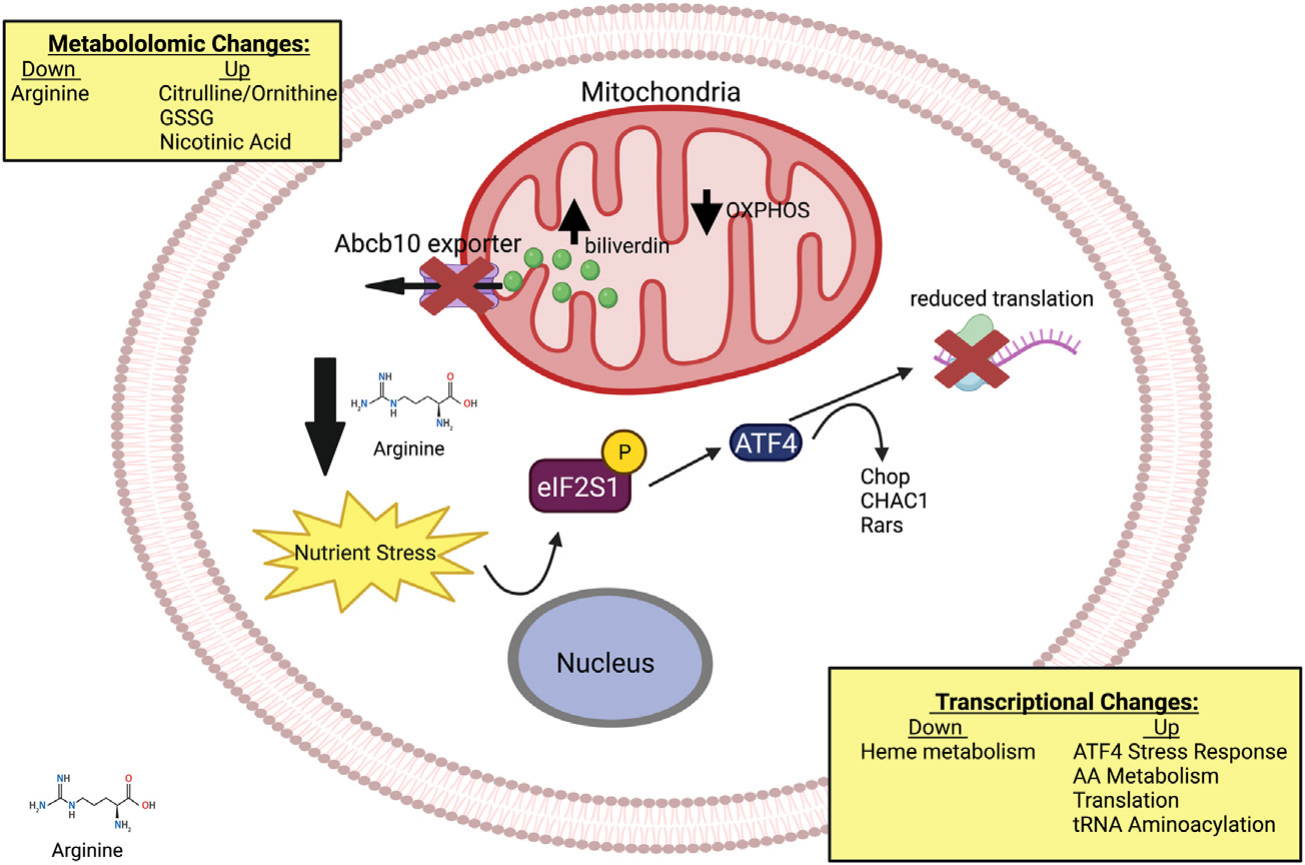
**Model of arginine metabolism shifts that signal nutrient stress in the absence of Abcb10.** Murine erythroleukemia (MEL) and human myelogenous leukemia (K562) cells lines can be differentiated to hemoglobinize. These cell lines have been utilized to inform on pathways necessary to induce heme synthesis. The absence of mitochondrial exporter Abcb10 in these model cell lines results in ineffective hemoglobinization. The alteration in mitochondrial homeostasis due to Abcb10 loss results in decreased oxidative phosphorylation (OXPHOS), increased cellular oxidative stress, and altered arginine metabolism. The changes give rise to nutrient stress signaling through eIF2S1 phosphorylation and activation of nutrient stress response transcriptional activator ATF4 and downstream targets that remodel transcription and block translation until nutrient stress is relieved. Providing increased arginine in culture medium alleviates the stress response and allows induction of hemoglobinization. eIF2S1, eukaryotic initiation factor-2S1.

Previous studies have shown Abcb10 is essential for life and that Abcb10 plays a key role in heme synthesis (6, 8, 11, 35). In the current study, we have confirmed the essential function of Abcb10 in heme production during erythroid differentiation. Our metabolomic analysis revealed a novel role for Abcb10 in arginine metabolism demonstrating a possible function for Abcb10 beyond heme synthesis. The loss of Abcb10 resulted in a significant diminution of arginine in both the cytosol and mitochondria of MEL cells even in undifferentiated cells, suggesting that Abcb10-specific mitochondrial exporter activity is required to maintain cellular amino acid homeostasis. Multiomic analyses revealed that Abcb10-null cells recognize the stress due to arginine depletion by phosphorylation of the translation initiation factor eIF2S1 during erythroid model MEL cell differentiation, which resulted in increased expression of *ATF4* and downstream targets *Chop, Chac1,* and *Rars* along with several other stress activated transcripts (Fig. 6). The increase in these stress response proteins alters the transcriptional profile of Abcb10-null cells during differentiation. The phosphorylation of eIF2S1 limits translation and cell proliferation, and the proliferation defects seen in both Abcb10-null K562 and MEL erythroleukemia cell lines could be recovered by increasing arginine levels in growth media. It is interesting to note that K562 cells were much more sensitive to arginine depletion even without loss of Abcb10. We do not know the reason for this increased arginine dependence but speculate that K562 cells are either less efficient at taking up arginine or regenerating arginine. Arginine tracing studies in MEL cells did not reveal any unusual utilization of arginine in the absence of Abcb10 making it difficult to determine why arginine metabolism is altered.

Perhaps, there is high arginine utilization for a specific protein made during hemoglobinization. During differentiation of erythroid precursors, the most prominent protein made is β-globin. Of the 147 amino acid in the β-globin chain, there are only three arginine residues, whereas there are >10 valines, 10 leucines, 8 histidines, 9 serines, as well as other amino acid making β-globin production not specifically arginine utilizing. Importantly, supplementation of other amino acids did not increase hemoglobinization in the absence of Abcb10 nor did supplementation of arginine metabolites citrulline and ornithine alter hemoglobinization in differentiating WT MEL or K562 cells.

One expectation of diminished arginine levels would be increased levels of arginine transporters such as CAT1 (36, 37). We observed a two-fold increase in expression of *CAT1* in Abcb10-null cells but did not see increased CAT1 protein. We also observed increased transcripts for several other proteins involved in arginine and proline metabolism as well as increased transcripts for a number of plasma membrane amino acids transporters (37, 38, 39, 40, 41). These transporters may be upregulated to compensate for the reduction in intracellular arginine in the absence of Abcb10. These results suggest that there is a transcriptional and translational response to loss of Abcb10.

Arginine limitation in mammalian cells has previously been shown to regulate translation. Indeed, loss of arginine tRNA charging leads to ribosomal pausing and ribosome pausing decreases protein synthesis and gives rise to premature ribosome termination while mRNA levels are unaltered (42). This suggests that in differentiating MEL cells that are limited for arginine, only a select set of transcripts are successfully translated. The finding of increased transcripts for tRNA aminoacylation supports that cells are trying to increase protein synthesis, and indeed, we observed increased *Rars* transcripts during differentiation in the absence of Abcb10, which equated to a 2- to 3-fold increase in Rars protein. We also saw increased transcripts for the nutrient stress sensing pathway ATF4 and downstream targets. Both *Chop* and *CHAC1* transcripts were increased 8- to 10-fold in the absence of Abcb10 during MEL cell differentiation, but protein levels for Chop and Chac1 either remained as in undifferentiated cells (Day 0) or increased only 2-fold. In contrast, WT cells showed decreases in *Chop* and *CHAC1* transcripts and protein during differentiation. Similarly, the transcripts and protein for the transcriptional sensory of nutrient limitation factor ATF4 decrease during differentiation in WT cells but stayed elevated in the absence of Abcb10. These results suggest that protein synthesis may be altered in the absence of Abcb10.

Hematopoietic stem cells rely on anaerobic glycolysis to maintain their inactive state and require a rapid shift from glycolysis to OXPHOS to meet the rigorous demands associated with differentiation (reviewed in (43)). This shift requires transcriptional remodeling. This can be mediated through epigenetic marks such as DNA methylation or histone modifications such as acetylation, methylation, or citrullination to name a few (reviewed in (44, 45)). These modifications require metabolites that are generated in the mitochondria. Several amino acids must be taken up or synthesized and transported into and out of the mitochondria including but not limited to glycine, glutamine, citrulline, and ornithine (46, 47, 48). Arginine has historically been considered a nonessential or semiessential amino acid, as it can be generated *de novo* from glutamine and proline (49). There are, however, certain metabolic states in which cellular biosynthesis of arginine is not sufficient for growth. In those instances, arginine must be acquired from exogenous sources. The inability to acquire sufficient arginine or synthesize intracellular arginine limits the capacity to make the transcriptional alterations necessary for effecting metabolic shifts depending upon nutrition and energy needs (28).

Arginine metabolism is known to be important in erythropoiesis (17, 18, 19). That we observed dramatically reduced levels of arginine in Abcb10-null cells with no difference in arginine uptake suggests that Abcb10 is involved in setting up the metabolic state necessary for proliferation and hemoglobinization. One readout for loss of Abcb10 in differentiating MEL cells is a loss in hemoglobinization. Previously, we demonstrated that siRNA-mediated Abcb10 knockdown using in MEL cells prevented BACH1 from coming off the *β-globin* promoter due to reduced heme synthesis blocking GATA1 chromatin access at the *β-globin* promoter (11). At that time, we did not appreciate that there were significant changes in metabolism when Abcb10 is lost. Our studies here show marked changes in arginine metabolism that give rise to nutritional stress, reduced energy production *via* decreased OXPHOS and glycolysis as measured by Seahorse analysis, and reduced protein translation and that arginine supplementation can partially relieve proliferation and hemoglobinization. We further identified that the enzymes ASS1 and ASL, which are responsible for intracellular arginine regeneration from citrulline (Fig. 2), are reduced in the absence of Abcb10 at both the transcriptional level and the protein level compared to WT cells. This suggests that these arginine regeneration steps may be rate limiting in the absence of Abcb10, and the metabolic tone set by the absence of Abcb10 limits intracellular arginine synthesis.

In our current study, we focused on arginine as it was the most dramatically changed amino acid with concomitant increases in citrulline and ornithine. Many studies have tied arginine homeostasis to red cell hemoglobinization (18, 20). Maeda *et al.* (18) determined that polyamines derived from arginine were important in proliferation and differentiation of both K562 and human cord blood-derived CD34-positive cells. Unfortunately, we did not detect polyamines in our metabolomic analyses, but we predict that the reduced levels of arginine in the absence of Abcb10 would give rise to reduce polyamine levels. In a very recent paper, Gonzalez-Menendez *et al.* (50) identified that arginine metabolism regulates erythroid differentiation through hypusination of the eukaryotic translation initiation factor 5A (eIF5A), which is done by conjugating spermidine to lysine on eIF5A. This study further determined that eIF5A hypusination was important for mitochondrial translation and that decreased hypusination diminished OXPHOS. We observed reduced OXPHOS activity in undifferentiated and differentiated Abcb10-null MEL cells. Our studies future advance our understanding of the role of Abcb10 in mitochondrial metabolism and erythroid differentiation. It is unclear if the change in Abcb10 substrate biliverdin localization causes a shift in arginine homeostasis. We attempted to address this question using hemoglobinization as a readout, however, providing exogenous bilirubin to MEL cells during differentiation reduced hemoglobinization even in WT cells and did not improve hemoglobinization in Abcb10-null cells making this analysis difficult to interpret. We speculate that the accumulation of biliverdin in the mitochondria affects the mitochondrial metabolic status. Further studies are needed to understand how the substrate transported by Abcb10 affects mitochondrial metabolism.

## Experimental procedures

### Tissue culture, plasmids, and transfections

MEL (DS19 clone), *Abcb10* CRISPR/CAS9 clone 12, and CRISPR/CAS9 clone J1 were maintained in Dulbecco’s Modified Essential Medium (DMEM) (Corning) containing 1.0 mM Na pyruvate (ThermoFisher), 1X Glutamax (ThermoFisher), 1X nonessential amino acids (Sigma-Aldrich), 10% fetal bovine serum, 100 units/ml penicillin, and 100 μg/ml streptomycin and incubated at 37 °C with 5% CO_2_. This MEL cell medium contained either 200% (1.68 g/l), 100% (0.84 g/l), 50% (0.42 g/l) or 5% (0.042 g/l) arginine. MEL cells were differentiated by incubating in culture media with 1.5% DMSO (Sigma-Aldrich) for 1 to 5 days in the presence or absence of 10 μM bilirubin. K562 WT, *ABCB10* CRISPR/CAS9 clones #13 and #40 cells were grown in RPMI1640, 1X Glutamax (ThermoFisher), 10% fetal bovine serum, 100 units/ml penicillin, and 100 μg/ml streptomycin and incubated at 37 °C with 5% CO_2_. K562 differentiation was done as described previously (51).

### Lentivirus production and transduction

CRISPR/CAS9 constructs were generated by the University of Utah Mutation Generation and Detection Core. CRISPR/CAS9 expression lentiviral vectors with GFP or BFP were packaged in HEK293T cells using a three-plasmid packaging system. The supernatant containing retroviruses was passed through 0.45 μm filter and stored in aliquots at −80 °C. Undifferentiated MEL cells (DS19) or K562 cells were transduced with 8 μg/ml of polybrene and CRIPSR lentiviruses that targeted exon 1 site 2 (E1S2) or exon 3 site 9 (E3S9) of the mouse *Abcb10* gene or exon 1 site 1 (E1S1) of the human *ABCB10* gene and grown for 2 days in growth media. Cells were sorted by flow cytometry for GFP- (mouse *Abcb10*) or BFP- (human *ABCB10*) positive cells, and single cells seeded into 96-well plates. Each construct generated several clones, and high melt resolution analysis was used to confirm changes in the target regions of *Abcb10*/*ABCB10* for each clone. Clones were identified for genomic sequencing, RT-qPCR, and Western blot.

### PCR and sequencing

Genomic DNA was isolated from WT and CRISPR/CAS9 MEL cells and K562 cells using the Invitrogen PureLink Genomic DNA Mini Kit. *Abcb10*/*ABCB10* was amplified from genomic DNA using Roche Expand Long Template PCR System, and primers used were as follows: mouse *Abcb10* exon 1, 5′-AGGAGAGCGAAAAAGCTTCC-3′ (forward), 5′-AGTCGTTCCCAAGGTCTCG-3′ (reverse), mouse *Abcb10* exon 3, 5′-GGCCACGTCACTACCTTCAT-3′ (forward), 5′-AGCAGAGGTGATGCCTTTGT-3′ (reverse), human *ABCB10* exon 1, 5′-ATGCGAGGCCCCCCTGCCT-3′ (forward), and 5′-GCATGGGACTCGCGGCCTC-3′ (reverse). 1.5% agarose gel in Tris/Borate/EDTA buffer (110.0 mM Tris; 90.0 mM Borate; 2.5 mM EDTA containing ethidium bromide) was run at 100 V for 1 h and imaged with Bio-Rad Image Lab Software to confirm PCR product at the correct molecular weight. PCR products were treated with ExoSAP-IT (ThermoFisher) prior to sequencing. Sequencing was performed by the University of Utah DNA Sequencing Core.

### RNA isolation, RNA-seq, and real-time quantitative PCR

RNA was extracted with the Direct-zol RNA/DNA miniprep kit (Zymo Research). Two μg of total RNA was used to synthesize cDNA using the High Capacity cDNA Reverse Transcription kit (AB Biosystems). Power SYBR Green Master mix (Life Technologies) was used on Realplex2 thermal cycler (Eppendorf). Actin was used as a control housekeeping gene. The ΔΔCt method was used to compare the variation of transcripts among samples. Specificity and efficiency were checked before using this method. Primer sequences are as follows: mouse *Actin*, 5′-GACGGCCAAGTCATCACTATTG-3′ (forward) and 5′CCACAGGATTCCATACCCAAGA 3′ (reverse); mouse *Abcb10*, 5′-ATGTACGCTTTCTGGGTTGG (forward) and 5′-TCCTGGAATACGGACACCTC-3′ (reverse); mouse *Alas2*, 5′-CTCCGAGGCATCTATGGCATC-3′ (forward) and 5′-ACACGAGGGTGTCTGCTTATG-3′ (reverse); mouse *Asl*, 5′-GGCAGAGACTAAAGGAGTGGCT-3′ (forward) and 5′-TCGACACTGGATTTCGCTGTGC-3′ (reverse); mouse NAPRT 5′-TTCCAGATGCCCTGGCTAGAGT-3′ (forward) and 5′-AGTGACCACACTGGTGCCAATG-3′ (reverse);mouse *Ass1*, 5′-ACTCAGGACCCTGCCAAAG-3′ (forward) and 5′-GCCATCTTTGATGTTGGTCA-3′ (reverse); mouse *Cat1*, 5-‘CTCCTGGCTTACTCTTTGGTGG-3′ (forward) and 5′-GATCTAGCTCCTCGGTGGTTCT-3′ (reverse), mouse *Padi3*, 5′-GTGGTCTTCGATTCTCCGAGGA-3′ (forward) and 5′- CAGGTTGCCAAAAGAGTCCAGAC-3′ (reverse), human *ABCB10* (forward) 5′-ATGACCGTGGGTGAACTCTC-3′ (reverse) 5′-CTCGTTAAAAGGCAGCTTGG-3′ and human *ACT* (forward) 5′-ATGGCCACGGCTGCTTCCAGC-3′, (reverse) 5′CATGGTGGTGCCGCCAGACAG-3′. For RNA-seq, total RNA samples (100–500 ng) were hybridized with Ribo-Zero Gold to substantially deplete cytoplasmic and mitochondrial rRNA from the samples. Globin Zero Gold kit (Illumina) was used to remove globin mRNA from total RNA. Read one and two adapter sequences were as follows: 5′-AGATCGGAAGAGCACACGTCTGAACTCCAGTCA-3′, and 5′-AGATCGGAAGAGCGTCGTGTAGGGAAAGAGTGT-3′. Stranded RNA sequencing libraries were prepared as described using the Illumina TruSeq Stranded Total RNA Library Prep Gold kit (20020613) with TruSeq RNA UD Indexes (20022371). Purified libraries were qualified on an Agilent Technologies 2200 TapeStation using a D1000 ScreenTape assay (cat# 5067-5582 and 5067-5583). The molarity of adapter-modified molecules was defined by quantitative PCR using the Kapa Biosystems Kapa Library Quant Kit (cat#KK4824). Individual libraries were normalized to 1.30 nM in preparation for Illumina sequence analysis. Sequencing libraries (1.3 nM) were chemically denatured and applied to an Illumina NovaSeq flow cell using the NovaSeq XP chemistry workflow (20021664). Following transfer of the flowcell to an Illumina NovaSeq instrument, a 2 × 51 cycle paired end sequence run was performed using a NovaSeq S1 reagent Kit (20027465). The mouse GRCm38 FASTA and GTF files were downloaded from Ensembl release 94, and the reference database was created using STAR version 2.6.1 b, as described (52) with splice junctions optimized for 50 base pair reads. Reads were trimmed of adapters using cutadapt 1.16 (53) and then aligned to the reference database using STAR in two pass mode to output a BAM file sorted by coordinates. Mapped reads were assigned to annotated genes in the GTF file using featureCounts, version 1.6.3, as described (54). The output files from cutadapt, FastQC, Picard CollectRnaSeqMetrics, STAR, and featureCounts were summarized using MultiQC (55) to check for any sample outliers. Differentially expressed genes were identified using a 5% false discovery rate with DESeq2 version 1.20.0 (56). Genes with multiple testing corrected *p* values <0.05 and an absolute log2FC >0.58 were used in GSEA (57) using the clusterProfiler package version 4.6.0 (58) in R (version 4.2.2) with Kyoto Encyclopedia of Genes and Genomes, Hallmark and Reactome gene sets. The RNA sequencing data have been deposited in the National Center for Biotechnology Information’s Gene Expression Omnibus under accession GSE225218. GSEA results for Days 0, two and five for Hallmark, Kyoto Encyclopedia of Genes and Genomes, and Reactome are provided in Table S1.

### Hemoglobinization

MEL and K562 cells were differentiated as previously described (11, 51). For arginine supplementation, normal DMEM arginine levels (0.4 mM) were increase to 2.0 mM or 2X normal growth medium. For bilirubin supplementation, cells were grown in 0.1% vehicle (DMSO) or 10 μM bilirubin in DMEM in undifferentiated and differentiated MEL cells for time.

### Ultra-performance liquid chromatography analysis of heme and porphyrins

MEL cells were differentiated for 0 to 96 h with 1.5% DMSO. Cells (20–50 × 10^6^) were pelleted, washed with sterile PBS, mixed with water to about 200 μl in a microcentrifuge tube, and sonicated for 12 cycles with 5 s intervals at 50% duty (about 2.5 s on, 2.5 s off) using a microtip. Alas2 activity of the resulting homogenate was determined as described previously (11). Briefly, washed and packed cell pellets were resuspended in approximately three volumes of 50 mM potassium phosphate (KPi) pH 7.4. The suspension was homogenized by a brief sonication using a Sonicare W-380 Ultrasonic Processor (Heat Systems-Ultrasonics, Inc), while in an ice bath. The protein content was measured by the Pierce BCA Protein Assay Method (Thermo Scientific) using a SpectraMax 190 microplate reader paired with SoftMax Pro v5.0b7 software, both from Molecular Devices Corp. Approximately, 25 μl of homogenate containing 10 mg/ml protein was mixed with 25 μl of assay buffer that contained 0.1 M glycine pH 7, 1 mM succinyl CoA, 160 μM pyridoxal 5′-phosphate, and 0.1 mM succinylacetone. The resulting mixture was incubated at 37 °C for 30 min, and then 450 μl of ice-cold water was added to stop the reaction. Fifty microliter of the stopped reaction was mixed with 150 μl derivatization agent that consisted of water, 37% formaldehyde, ethanol, and acetylacetone in a ratio of 107:5:15:23 by volume, respectively. The resulting solution was incubated at 100 to 103 °C for 5 min, cooled immediately in an ice bath for a minimum of 1 h in the dark, and then centrifuged for 10 min at 16,000*g* at 4 °C. The supernatant was analyzed by ultra-performance liquid chromatography (UPLC). Ten microliter of supernatant containing the derivatized ALA was injected into a Waters Acquity UPLC system which included a binary solvent manager, sample manager, fluorescence detector, column heater, and an Acquity UPLC BEH C18, 1.7 μM, 2.1 Å 100 mm column. The fluorescence detector was set at 370 nm excitation and 460 nm emission, while the sample chamber was kept dark and at 5 °C. Solvent A was 0.2 % aqueous formic acid, while solvent B was 100% methanol. The flow rate was constant at 0.3 ml/min, and the column at 50 °C for the total run time of 12 min. Blanks were samples heated in boiling water a bath for 10 min. Standard curves were constructed using samples spiked with known amounts of ALA.

Total intermediate porphyrins (8-, 7-, 6-, 5-, and 4-carboxyl porphyrins) were measured in the cell homogenate previously adjusted to 10 mg/ml protein by adding 3 M HCl to a final concentration of 1.5 M, incubated for 0.5 h at 37 °C in a water bath, and microfuged at maximum speed for 10 min. About 10 μl of the supernatant was injected into the same Waters UPLC system mentioned above and quantified fluorometrically at 404 nm excitation and 618 nm emission.

Analyses of heme and protoporphyrin IX were carried out by vigorously mixing a 50-μl aliquot of 10 mg/ml homogenate was with 200 μl of an extraction mixture of ethyl acetate (4 volumes) and glacial acetic acid (1 volume). The phases were separated by microcentrifugation for 1 min at maximum speed. The upper organic layer was immediately analyzed simultaneously for protoporphyrin IX fluorescence at 404 nm excitation and 630 nm emission and heme absorbance at 398 nm in the same UPLC system mentioned above and as previously described (11, 59).

### Metabolite extraction from mitochondria for LC-MS analysis

One hundred microliters of a solution of 9:1 cold ACN:ddH2O containing 0.1 μg/ml standards of carnitine-d9, TMAO-d9, and succinate-d4 was added to each MEL cell mitochondrial pellet. A process blank containing only extraction solvent and standards was created and carried through the extraction process. Samples were vortexed for 30 s prior to being chilled for 1 h at −20 °C. After 1 h at −20 °C, samples were centrifuged at 20,000*g*, and the supernatant was collected for untargeted LC-MS analysis. A pooled QC was created by combining 5 μl of each sample of their respective extraction. For cytosol, 100 μl of a solution of 9:1 cold ACN:ddH_2_O containing 0.1 μg/ml standards of carnitine-d9, TMAO-d9, and succinate-d4 was added to 43 μl of cytosol or media. A process blank containing only extraction solvent, 43 μl ddH_2_O, and standards was created and carried through the extraction process. Samples were vortexed for 30 s prior to being chilled for 1 h at −20 °C. After 1 h at −20 °C, samples were centrifuged at 20,000*g*, and the supernatant was collected for untargeted LC-MS analysis. A pooled QC was created by combining 5 μl of each sample of their respective extraction.

### Untargeted LC-MS analysis

Fifty microliters of each sample, QC, and process blank solution were added to individual PTFE auto sampler vials and randomized prior to analysis. An Agilent 1290 UPLC coupled to an Agilent 6545 Q-ToF (Agilent Technologies, Inc) was used for chromatography and mass spectrometry. Chromatography was performed with a Waters BEH amide 2.1 × 100 mm column with BEH amide precolumn (Waters Corporation). An initial concentration of 99% buffer A (ACN with 5% 10 mM NH4OAc ddH2O) and 1% buffer B (10 mM NH4OAc in ddH2O) was held for 1.66 min at a flow rate of 0.4 ml/min. B was decreased to 70% over 4.75 min then to 40% over 1.5 min. Finally, B was decreased to 30% over 0.68 min and held for 3.75 min. The system was allowed to re-equilibrate for 5.16 min between runs. Samples were analyzed by an Agilent 6545 with a Dual Jetstream ESI source in positive and negative modes with the following source parameters: Gas Temp = 275 °C, Drying Gas = 12 l/min, Nebulizer = 35 psig, Sheath Gas Temp 325 °C, Sheath Gas Flow 12 l/min, VCap = 3500 V, Nozzle Voltage = 250 V, Fragmentor = 100 V, Skimmer = 65 V. Data analysis was performed using Agilent Profinder and Quantitative Analysis software (Agilent Technologies, Inc).

### Targeted LC-MS analysis

Mass spectral analysis was performed using a SCIEX EXION LC system coupled to a SCIEX 6500 Q-Trap (Sciex). A Waters BEH amide 2.1 × 100 was used to separate lysine and citrulline using the same chromatography method as above. To separate ornithine and arginine, helicon iHILIC-Fusion 2.1 × 100 mm (Umeå) was used for chromatography using Buffer C (10 mM NH4OAc in water) and ACN. An initial concentration of 85% ACN was held for 1 min then decreased to 10% until 11 min, then returned to initial conditions over 1 min. The column was allowed to equilibrate for 11 min between runs. Mass spectrometry was performed in the positive mode with a TurboIon source using optimized source conditions: curtain gas = 20 l/min, collision gas = high, ion spray voltage = 5000 V, temperature = 400 °C, ion source gas 1 = 20 l/min, ion source gas 2 = 40. Declustering potential, entrance potential, and exit potential for all compounds were set to 50 V, 10 V, and 15 V, respectively. Transitions for d9-carnitine (171.1 -> 103.1, CE = 23 V), citrulline (176.1 -> 159.1, CE = 10 V, 176.1 -> 70.1, CE = 10 V, and 176.1 -> 113.1, CE = 10 V), lysine (147.1 -> 84.1, CE = 10, 147.1 -> 130.1, CE = 10), ornithine (133.1 -> 70.1, CE = 10 V, 133.1 -> 116.1, CE = 10 V), and arginine (171.1 -> 60.1, CE = 10, 171.1 -> 116.1, CE = 10 V, 171.1 -> 70.1 CE = 20 V) were monitored *via* MRM. Quantitative data analysis was conducted using Sciex MultiQuant software.

### Nitric oxide quantitation

MEL cells were differentiated for 0, 2, and 5 days. Five million cells were harvested and washed twice with Hank’s Balanced Salt Solution (HBSS) (140 mM sodium chloride, 5 mM potassium chloride, 1 mM calcium chloride, 400 μM magnesium sulfate, 500 μM magnesium chloride, 300 μM sodium phosphate, 400 μM potassium phosphate, 6 mM glucose, 4 mM sodium bicarbonate). Cells were incubated with 2 μM of DAF-FM (Invitrogen) for 20 min at 37 °C and washed twice with HBSS followed by a second incubation with HBSS for the same duration and temperature. Cells were transferred to a 96-well plate and the BioTek Synergy 4 was set to measure fluorescence at 495 nm excitation and 515 nm emission. Background fluorescence was measured with cells that were not exposed to DAF-FM at the same excitation and emission.

### Arginine uptake

Control (mCherry) and *Abcb10* disrupted MEL cells (2 × 10^5^) were incubated in the presence of 25 μM ^3^H-arginine in (−) arginine DMEM and uptake measured at 37 °C over time (0–10 min). Cells were washed with 1 ml of ice-cold PBS 4x and cell pellets resuspended in 1 ml of water and counted for radioactivity in liquid scintillation cocktail (Packard Instrument Co). Nonspecific cell associated ^3^H-arginine, uptake done at 0 °C was averaged and subtracted as background. Assays were run with a minimum of five experimental replicates.

### MitoSOX

Control and *Abcb10* disrupted MEL cells were differentiated as described and incubated in 5 μM MitoSOX in Hank’s balanced salts solution for 10 min at 37 °C, washed twice, and resuspended in 1 ml buffer prior to fluorescence detection as per manufacturer’s instructions.

### Seahorse analysis

Control (mCherry) and *Abcb10* disrupted MEL cells were differentiated as described (eight replicates) and 1 × 10^5^ cells per well seeded, centrifuged at 1500*g* for 10 min, and incubated for 20 to 60 min 37 °C in a non-CO_2_ incubator. Following initial incubation, XF Running Media (XF assay media with 5% FCS and 10 mM D-glucose) were dispensed into each well. Oxygen consumption rate and extracellular acidification rate were measured by an XF96 Seahorse Extracellular Flux Analyzer. Cells were treated with oligomycin (1.0 μM), carbonyl cyanide 4-(trifluoromethoxy)phenylhydrazone (1.5 μM), rotenone (100 nM), and antimycin A (1.0 μM) as per manufacturer’s instructions. Each condition was performed in 2 to 3 technical replicates.)

### Other procedures

Crude mitochondria and cytosol were isolated from MEL cells using a mitochondrial isolation kit (Pierce). MEL cell mitochondria or whole cells were lysed in buffer (150 mM NaCl, 1% Triton, 10 mM EDTA, and 10 mM Trizma base) with 2X protease inhibitors (Roche) and incubated at 0 °C for 30 min, followed by centrifugation at 12,000*g* for 15 min. The supernatants (10–50 μg) were placed in Laemmli buffer and heated to either 65 °C for 20 min or 100 °C for 10 min. Samples were run on a 4 to 20% SDS-PAGE gel (Bio-Rad) at 100 V for 60 to 90 min in buffer (25 mM Tris, 192 mM glycine, 0.1% SDS) and transferred to nitrocellulose membranes (GE Healthcare Life Sciences) at 100 V for 1 h in Western transfer buffer (25 mM Tris, 192 mM glycine, 20% (v/v) methanol). Primary antibodies Abcb10 (rabbit, 1:1000; ThermoFisher Scientific, PA5-30468, RRID AB_2547942), CAT1 (rabbit, 1:500; LifeSpan BioSciences, LS-C749764), ASS (rabbit, 1:500; Sigma-Aldrich, AV41366), ASL (rabbit 1:500; ThermoFisher Scientific, PA5-22300, RRID AB_11152934), VDAC (rabbit, 1:1000; Abcam, ab154856), Actin (mouse, 1:1000; ThermoFisher MA1-744, RRID AB_2223496), ATF4 (rabbit, 1:1000; ThermoFisher MA5-32364, RRID AB_2809645), CHAC1 (rabbit, 1:500; ThermoFisher BS-6795R), CHOP (rabbit, 1:500; ThermoFisher PA5-104528, RRID AB_2853828), eIF2s1 (rabbit, 1:500, ThermoFisher AHO1182, RRID AB_2536326) and eIF2s1Phos (rabbit, 1:500, ThermoFisher MA5-15133, RRID AB_10983400), RARS (rabbit, 1:500 ThermoFisher PA5-110271,RRID AB_2855682), and Slc7a11 (rabbit, 1:500 ThermoFisher PA 1–16893, RRID AB_2286208) were incubated overnight at 4 °C. Membranes were washed extensively and incubated in a 1:5000 dilution of peroxidase-conjugated goat anti-rabbit or anti-mouse at 37 °C for 60 min. Western blots were developed using Western Lighting reagent (PerkinElmer). Blots were quantified using Fiji ImageJ software using Actin as a loading control.

### Statistical analysis

Data are expressed as mean ± SD from at least three independent experiments unless stated otherwise. Statistical analyses were performed using student’s *t* test or two-way ANOVA as appropriate with GraphPad Prism software. Outliers were identified in Prism using ROUT (Q = 1.0%). *p* value of less than 0.05 was considered statistically significant and was indicated by asterisks; ∗*p* ≤ 0.05, ∗∗*p* ≤ 0.01, ∗∗∗*p* ≤ 0.001, ∗∗∗∗*p* ≤ 0.0001.

## Data availability

All data are contained within the manuscript.

## Supporting information

This article contains supporting information.

## Conflict of interest

The authors declare no conflicts of interest with the contents of this article.
