# Supplementary material for: Loss of the mitochondrial protein Abcb10 results in altered arginine metabolism in MEL and K562 cells and nutrient stress signaling through ATF4
Source: J Biol Chem. 2023 Jun 1;299(7):104877. doi: 10.1016/j.jbc.2023.104877 (PMC10316008; doi:10.1016/j.jbc.2023.104877)
Supplement: Supporting Figures S1–S4 and Table S1 [file mmc1.pdf]

Loss of the mitochondrial protein Abcb10 results in altered arginine metabolism in MEL and K562 cells and nutrient stress signaling through ATF4

Marisa Miljkovics[1], Alexandra Sequin[1], Xuan Jia[1], James E. Cox[2,3], Jonathan Leon Catrow[3], Hector Bergonia[4], John D. Phillips[5], W. Zac Stephens[1] and Diane M. Ward[1,6]

Supplemental Information Included

Figure Legends S1-4

Figures S1-4

Table S1 Legend

Table S1

SFigure 1 Abcb10 CRISPR/Cas9 MEL cell clones show incomplete hemoglobinization

A) WT, Clone 12 (complete null E1S2) and Clone J (partial null E3S9) cells were hemoglobinized for 0-5 days as described in Materials and Methods. (n$\geq$3 biologic replicate/experiment; 3 experiments). A representative Western blot for Abcb10 and mitochondrial loading control VDAC is shown. Note the slight hemoglobinization in clone J at day 5 of differentiation along with a low level of Abcb10 protein upon differentiation. B) RNA Seq for WT and Abcb10 null (Clone 12) was performed on cells as in A. Transcripts for *Alad*, *Urod*, *Cpox*, *Ppox*, *Uros* and *Fech* are shown as FPKM (n=5 biologic replicates). Bar graphs show the mean +/- SD with Student's *t* test significance $p\leq0.05$.

SFigure 2 Alterations in metabolites due to Abcb10 loss

A) GSSG and GSH levels were determined from targeted metabolomics as in Figure 2A. Bar graphs show the mean +/- SD with Student's *t* test significance $p\leq0.05$, (n=5-6 replicates with 2 biologically separate metabolomics performed). B) Cytosolic and Mitochondrial Pyruvate level were measured as in 2A. Bar graphs show the mean +/- SD with Student's *t* test significance $p\leq0.05$, (n=5-6 replicates with 2 biologically separate metabolomics performed). C) Orotic acid and creatine levels in cytosol and mitochondria were determined from metabolomics as in Figure 2A. Bar graphs show the mean +/- SD with Student's *t* test significance $p\leq0.05$, (n=5-6 replicates with 2 biologically separate metabolomics performed). D) Proline and glutamate levels from 2A are graphed. Bar graphs show the mean +/- SD with Student's *t* test significance

$p \leq 0.05$, (n=5-6 replicates with 2 biologically separate metabolomics performed). The data are expressed as Arbitrary units in A-D. E) Transcript levels for *Padi3* with *Actin* as a loading control were performed using RT-qPCR as described in Materials and Methods. Bar graphs show the mean +/- SD with Student's *t* test significance $p \leq 0.05$ (n=2 technical replicates and 5 biologic replicates).

SFigure 3 Loss of Abcb10 slightly alters arginine uptake in MEL cells

A) WT or Abcb10 null cells were incubated with ^{3}H-arginine and cell associated ^{3}H-arginine determined over time. (n=3 biologic replicate experiments). Two way ANOVA with significance $p \leq 0.05$ * .B) RNA Seq from undifferentiated and differentiated WT and Abcb10-null MEL cells was performed as described in Materials and Methods. Transcript levels (FPKM) for *Slc7a5, Slc7a6, Slc7a7* and *Slc7a11* are shown. Bar graphs show the mean +/- SD with Student's *t* test significance $p \leq 0.05$. C) WT or Abcb10-null cells were differentiated for time in the presence or absence of 10 µM bilirubin, cells pelleted and washed extensively in PBS to eliminate extracellular bilirubin. Cell pellets were image with duplicate technical samples shown (n=3 biologic replicate experiments). D) Heme and bilirubin levels were measure in cells as in C. Bar graphs show the mean +/- SD with Student's *t* test significance $p \leq 0.05$.

SFigure 4 Aspartate supplementation to Abcb10-null MEL cells does not improve hemoglobinization

A) WT and Abcb10 null MEL cells were differentiated for 0 or 5 days as described in Materials and Methods in the presence or absence of 2X arginine (arg), aspartate (asp) or both arg and asp. Redness in cell pellets denotes hemoglobinization. B) Extracellular Acidification Rate (ECAR), a readout of glycolytic activity was measured in cells as in Figure 4. C) Cells as in A were differentiated for 0, 2, or 5 days and incubated with the superoxide indicator MitoSOX as described in Materials and Methods (n=3 biologic replicates experiments). Bar graphs show the mean +/- SD with Student's *t* test significance $p \leq 0.05$

STable 1 GSEA results comparing WT and Abcb10 nulls cells at day 0-5 of differentiation

RNA seq GSEA from cells as in Figure 5 are shown for days 0-5 of differentiation. Hallmark, Kegg and Reactome results are shown.

Supplemental Figure 1

A

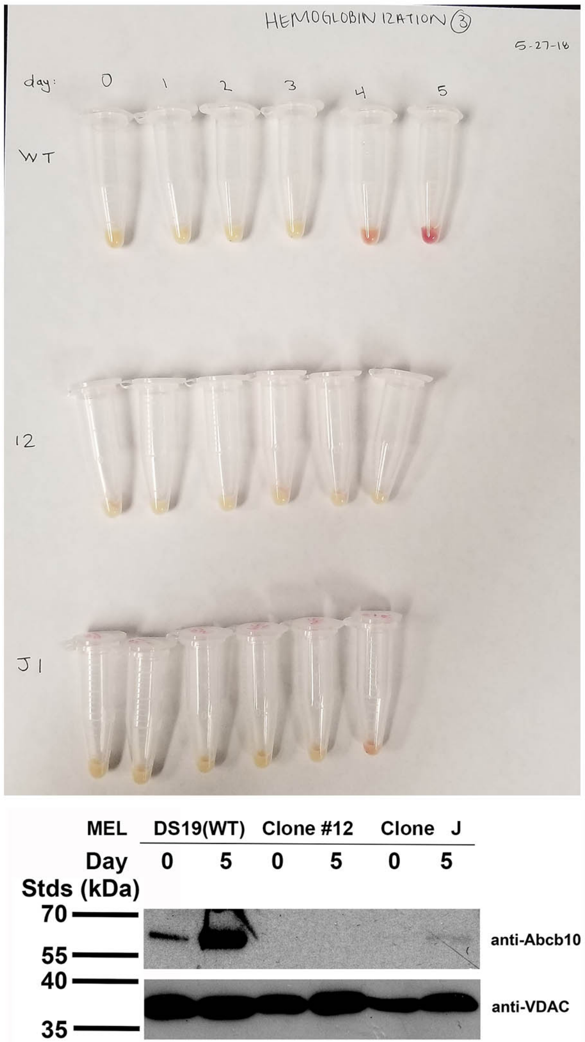

B

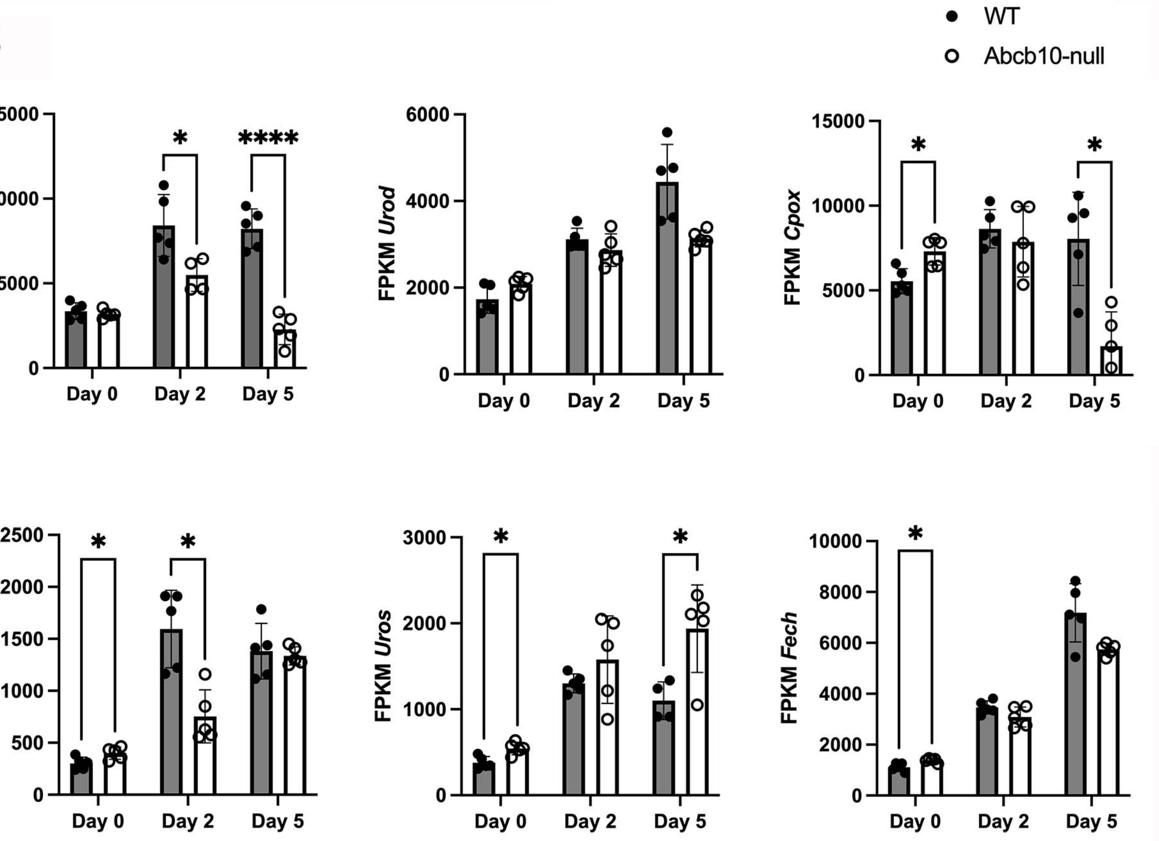

Supplemental Figure 2

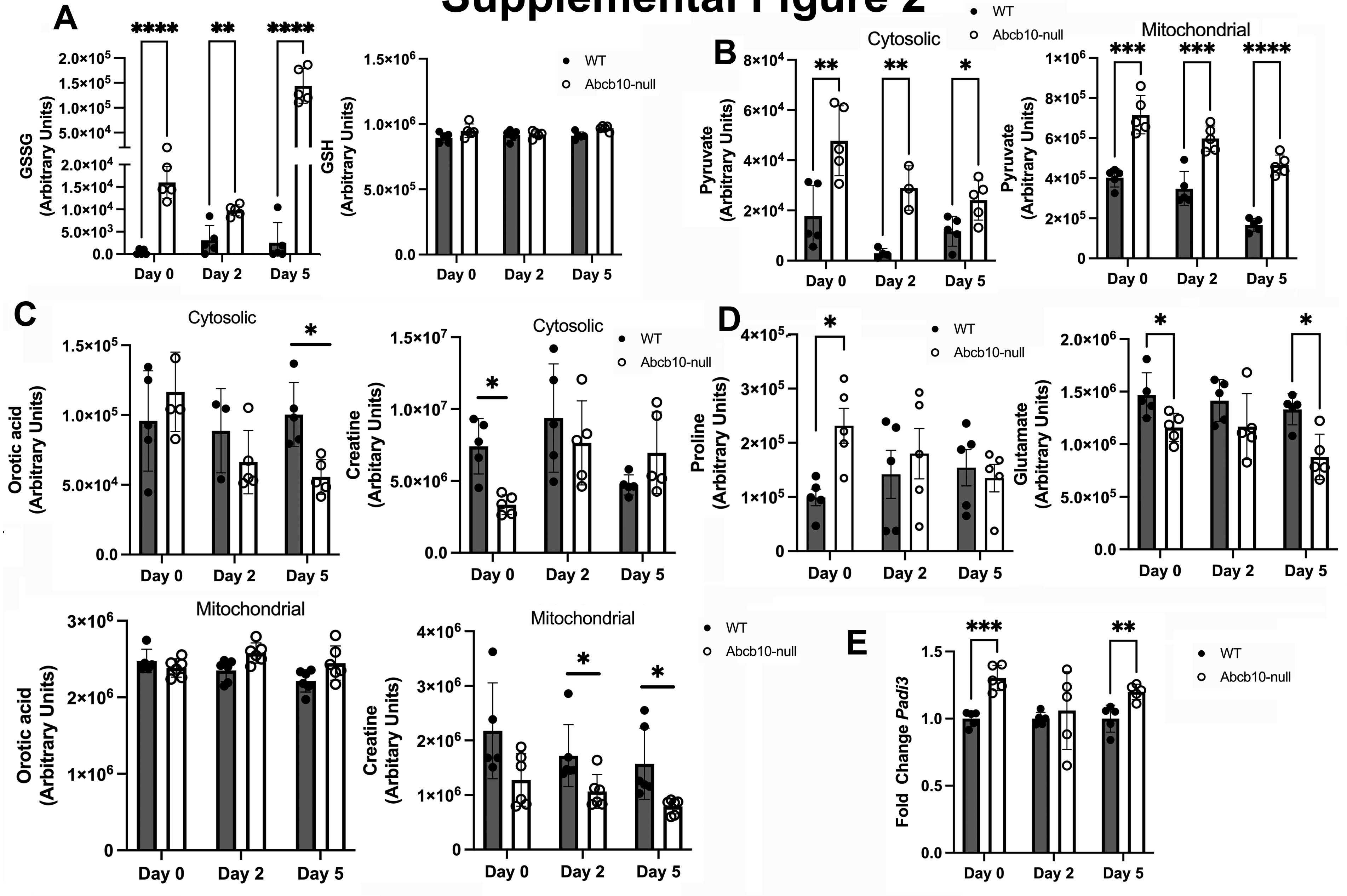

Supplemental Figure 3

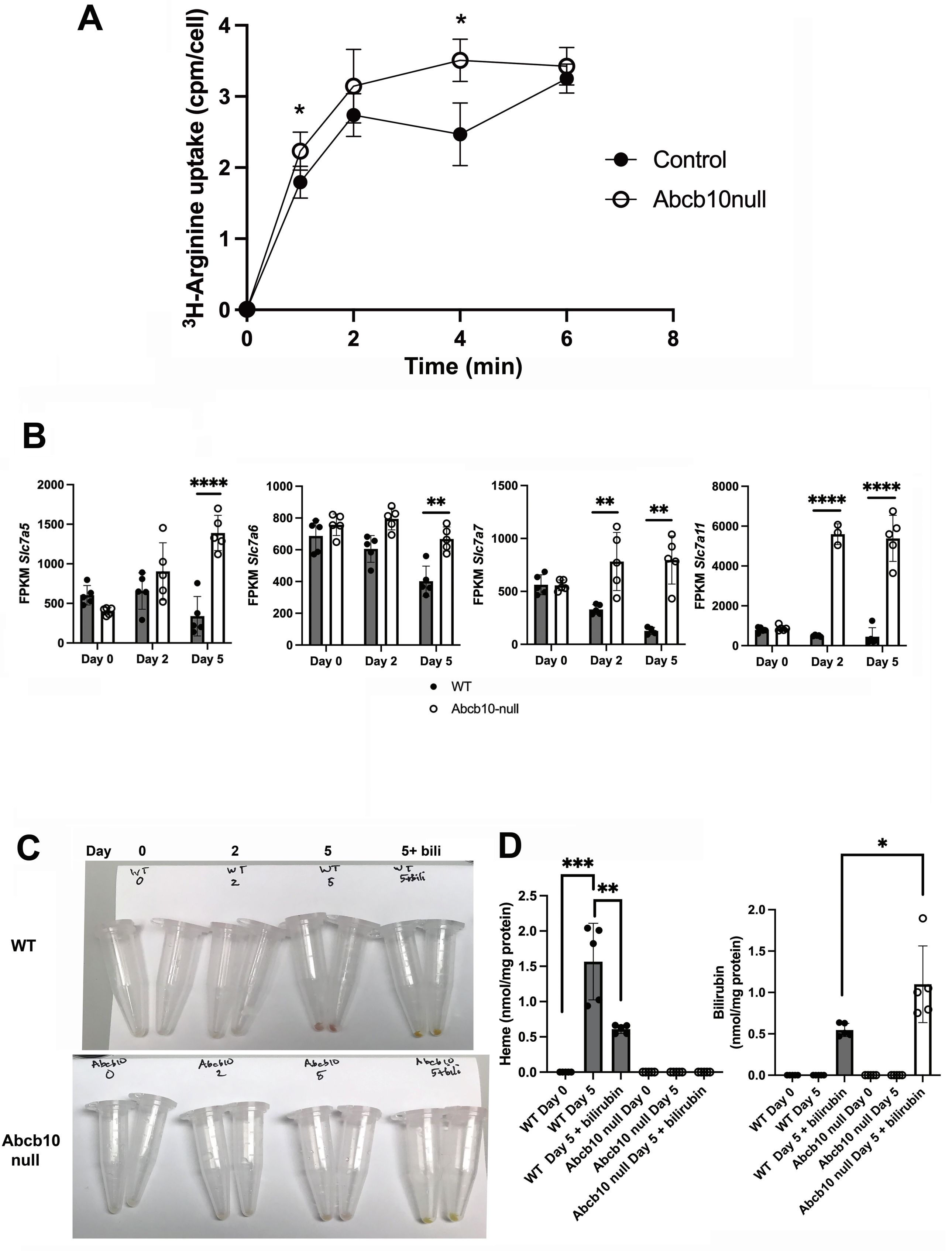

Supplemental Figure 4

A

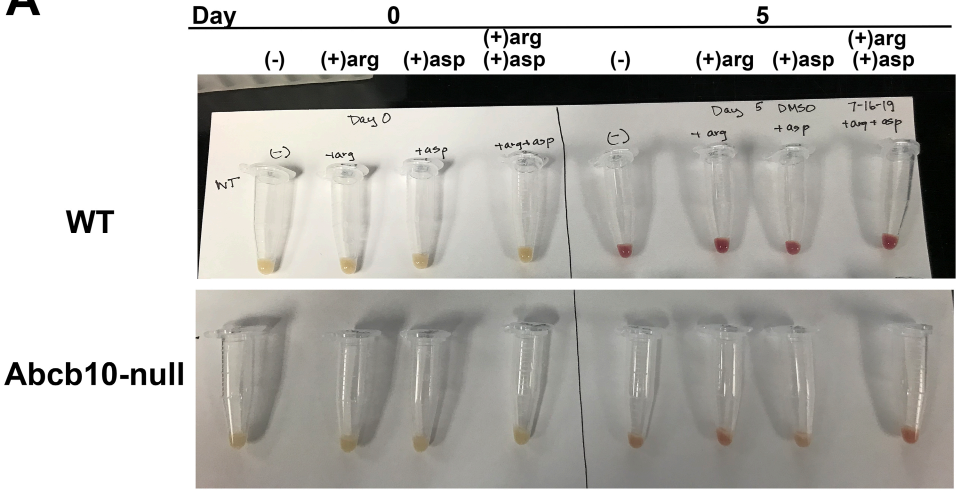

C

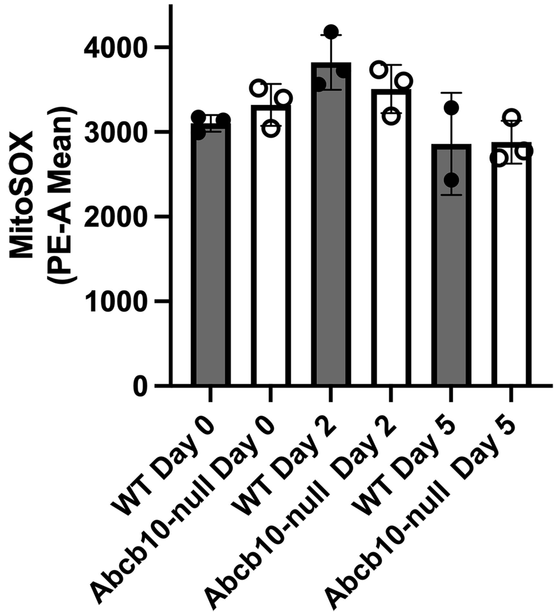

B

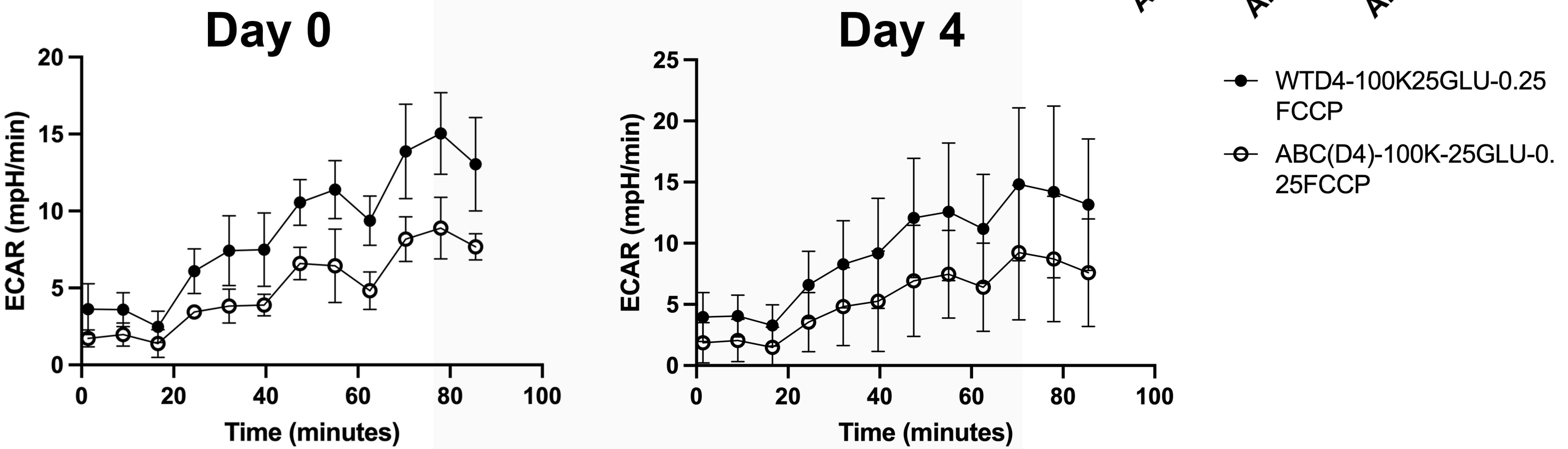

ID Day0	p.adjust	NES	core_enrichment
HALLMARK_INTERFERON_GAMMA_RESPONSE	4.8777E-11	2.83341069	Rsad2/Irf7/Xaf1/Rtp4/Lgals3bp/Usp18/Epsti1/Rigi/Mx2/Isg15/Ddx60/Dhx58/Cmpk2/Ifit1bl1/Rnf213/Gbp3/Gbp9/Oas2/Oas3/Samd9l/Parp12/Parp14/Irf9/Casp4/Ifih1/Nod1/Trim21/Herc6/Ube2l6/Psmb9/Ifi35/Oasl1/Sp110/Eif2ak2/Ifi27/Tdrd7/Trim14/Isg20/Tap1/Il10ra/Tapbp/Bst2/H2-Q10/Nmi
HALLMARK_INTERFERON_ALPHA_RESPONSE	1.9753E-10	2.81760807	Oas1g/Rsad2/Irf7/Rtp4/Lgals3bp/Usp18/Epsti1/Mx2/Isg15/Ddx60/Dhx58/Gbp2/Cmpk2/Gbp3/Parp9/Oas1a/Samd9l/Parp12/Parp14/Irf9/Trim12c/Ifih1/Trim21/Herc6/Uba7/Ube2l6/Psmb9/Ifi35/Oasl1/Sp110/Eif2ak2/Ifi27/Tdrd7/Trim14/Isg20/Tap1/Bst2/H2-Q10/Nmi/Tmem140/Psmb8
HALLMARK_ALLOGRAFT_REJECTION	0.03447432	1.8618203	Irf7/Gzmb/Gbp2/Ccr1/H2-T23/F2/Tlr6/Tap1/Tapbp/H2-Q10
HALLMARK_COMPLEMENT	0.03447432	1.83628714	Irf7/Gzmb/Ctso/Lgals3/Dpp4/F2/Casp4/Psmb9/Cd55/Fyn/Gnb4/Casp3/Dock10/Lipa/Irf1
KEGG_SYSTEMIC_LUPUS_ERYTHEMATOSUS	5.0716E-05	-2.5971622	H4c6/H2ac4/H2bc3/Snrpd3/H2ac6/H3c4/H4c4/H3c2/H3c11/H2ac22/H4c18/H3c3/H2ac21/H2ac12/H2ac7/H2ac13/H2ac8/H2ac10/H4c1/H4c3
REACTOME_INTERFERON_SIGNALING	8.6366E-07	2.59636433	Oas1g/Rsad2/Irf7/Xaf1/Trim34a/Usp18/Rigi/Mx2/Isg15/Gbp2/Ifit1bl1/Gbp3/Gbp9/Oas2/Oas3/Oas1a/Gbp7/Sp100/H2-T23/Irf9/Trim12c/Trim21/Uba7/Ube2l6/Ifi35/Oasl1/Eif2ak2/Ifi27
REACTOME_CYTOKINE_SIGNALING_IN_IMMUNE_SYSTEM	4.9333E-06	2.43443429	Oas1g/Rsad2/Irf7/Xaf1/Trim34a/Usp18/Rigi/Mx2/Isg15/Gbp2/Ifit1bl1/Gbp3/Gbp9/Oas2/Oas3/Ccr1/Oas1a/Gbp7/Sp100/H2-T23/Irf9/Trim12c/Nod1/Tec/Trim21/Uba7/Ube2l6/Psmb9/Ifi35/Oasl1/Eif2ak2/Ifi27/Trim14/Isg20/Il1rl1/Fyn/Il10ra/Bst2/H2-Q10
REACTOME_HCMV_LATE_EVENTS	1.2878E-05	-2.8349782	H2ac4/H2bc3/H2ac6/H3c4/H4c4/H3c2/H3c11/H2ac22/H4c18/H3c3/H2ac21/H2ac12/H2ac7/H2ac13/H2ac8/H2ac10/H4c1/H4c3
REACTOME_HDACS_DEACETYLATE_HISTONES	1.2878E-05	-2.8349782	H2ac4/H2bc3/H2ac6/H3c4/H4c4/H3c2/H3c11/H2ac22/H4c18/H3c3/H2ac21/H2ac12/H2ac7/H2ac13/H2ac8/H2ac10/H4c1/H4c3
REACTOME_ANTIVIRAL_MECHANISM_BY_IFN_STIMULATED_GENES	1.2878E-05	2.4325498	Oas1g/Usp18/Rigi/Mx2/Isg15/Ifit1bl1/Oas2/Oas3/Oas1a/Uba7/Ube2l6/Oasl1/Eif2ak2
REACTOME_RMTS_METHYLATE_HISTONE_ARGININES	2.7358E-05	-2.7605854	H2ac4/H2ac6/H3c4/H4c4/H3c2/H3c11/H2ac22/H4c18/H3c3/H2ac21/H2ac12/H2ac7/H2ac13/H2ac8/H2ac10/H4c1/H4c3
REACTOME_INTERFERON_ALPHA_BETA_SIGNALING	2.7358E-05	2.43722247	Oas1g/Rsad2/Irf7/Xaf1/Usp18/Mx2/Isg15/Gbp2/Ifit1bl1/Oas2/Oas3/Oas1a
REACTOME_HATS_ACETYLATE_HISTONES	3.7233E-05	-2.7651312	H4c6/H2ac4/H2bc3/H2ac6/H3c4/H4c4/H3c2/H3c11/H2ac22/H4c18/H3c3/H2ac21/H2ac12/H2ac7/H2ac13/H2ac8/H2ac10/H4c1/H4c3
REACTOME_HCMV_EARLY_EVENTS	6.6097E-05	-2.7144118	H4c6/H2ac4/H2bc3/H2ac6/H3c4/H4c4/H3c2/H3c11/H2ac22/H4c18/H3c3/H2ac21/H2ac12/H2ac7/H2ac13/H2ac8/H2ac10/H4c1/H4c3
REACTOME_HCMV_INFECTION	6.6097E-05	-2.7144118	H4c6/H2ac4/H2bc3/H2ac6/H3c4/H4c4/H3c2/H3c11/H2ac22/H4c18/H3c3/H2ac21/H2ac12/H2ac7/H2ac13/H2ac8/H2ac10/H4c1/H4c3
REACTOME_FORMATION_OF_THE_BETA_CATENIN_TCF_TRANSACTIVA	0.00014139	-2.5369859	H2ac4/H2bc3/Tle1/H3c4/H4c4/H3c2/H3c11/H4c18/H3c3/H2ac7/H2ac13/H2ac10/H4c1/H4c3
REACTOME_CELLULAR_SENESCENCE	0.00015029	-2.5395235	Ube2c/H4c6/H2ac4/Ube2s/Terf2ip/H2bc3/H3c4/H4c4/H3c2/H3c11/H4c18/H3c3/H2ac7/H2ac13/Id1/H2ac10/H4c1/H4c3
REACTOME_CHROMATIN_MODIFYING_ENZYMES	0.00015029	-2.5383422	H4c6/H2ac4/H2bc3/H2ac6/H3c4/H4c4/H3c2/H3c11/H2ac22/H4c18/H3c3/H2ac21/H2ac12/H2ac7/H2ac13/H2ac8/H2ac10/H4c1/H4c3
REACTOME_ACTIVATED_PKN1_STIMULATES_TRANSCRIPTION_OF_AR	0.00015029	-2.511161	H2ac4/H2bc3/H3c4/H4c4/H3c2/H3c11/H4c18/H3c3/H2ac7/H2ac13/H2ac10/H4c1/H4c3
REACTOME_ASSEMBLY_OF_THE_ORC_COMPLEX_AT_THE_ORIGIN_OI	0.00015029	-2.511161	H2ac4/H2bc3/H3c4/H4c4/H3c2/H3c11/H4c18/H3c3/H2ac7/H2ac13/H2ac10/H4c1/H4c3
REACTOME_B_WICH_COMPLEX_POSITIVELY_REGULATES_RRNA_EXP	0.00015029	-2.511161	H2ac4/H2bc3/H3c4/H4c4/H3c2/H3c11/H4c18/H3c3/H2ac7/H2ac13/H2ac10/H4c1/H4c3
REACTOME_CONDENSATION_OF_PROPHASE_CHROMOSOMES	0.00015029	-2.511161	H2ac4/H2bc3/H3c4/H4c4/H3c2/H3c11/H4c18/H3c3/H2ac7/H2ac13/H2ac10/H4c1/H4c3
REACTOME_DISEASES_OF_PROGRAMMED_CELL_DEATH	0.00015029	-2.511161	H2ac4/H2bc3/H3c4/H4c4/H3c2/H3c11/H4c18/H3c3/H2ac7/H2ac13/H2ac10/H4c1/H4c3
REACTOME_DNA_METHYLATION	0.00015029	-2.511161	H2ac4/H2bc3/H3c4/H4c4/H3c2/H3c11/H4c18/H3c3/H2ac7/H2ac13/H2ac10/H4c1/H4c3
REACTOME_EPIGENETIC_REGULATION_OF_GENE_EXPRESSION	0.00015029	-2.511161	H2ac4/H2bc3/H3c4/H4c4/H3c2/H3c11/H4c18/H3c3/H2ac7/H2ac13/H2ac10/H4c1/H4c3
REACTOME_ERCC6_CSB_AND_EHMT2_G9A_POSITIVELY_REGULATE_	0.00015029	-2.511161	H2ac4/H2bc3/H3c4/H4c4/H3c2/H3c11/H4c18/H3c3/H2ac7/H2ac13/H2ac10/H4c1/H4c3
REACTOME_GENE_SILENCING_BY_RNA	0.00015029	-2.511161	H2ac4/H2bc3/H3c4/H4c4/H3c2/H3c11/H4c18/H3c3/H2ac7/H2ac13/H2ac10/H4c1/H4c3
REACTOME_NEGATIVE_EPIGENETIC_REGULATION_OF_RRNA_EXPRES	0.00015029	-2.511161	H2ac4/H2bc3/H3c4/H4c4/H3c2/H3c11/H4c18/H3c3/H2ac7/H2ac13/H2ac10/H4c1/H4c3
REACTOME_OXIDATIVE_STRESS_INDUCED_SENESCENCE	0.00015029	-2.511161	H2ac4/H2bc3/H3c4/H4c4/H3c2/H3c11/H4c18/H3c3/H2ac7/H2ac13/H2ac10/H4c1/H4c3
REACTOME_POSITIVE_EPIGENETIC_REGULATION_OF_RRNA_EXPRESS	0.00015029	-2.511161	H2ac4/H2bc3/H3c4/H4c4/H3c2/H3c11/H4c18/H3c3/H2ac7/H2ac13/H2ac10/H4c1/H4c3
REACTOME_PRC2_METHYLATES_HISTONES_AND_DNA	0.00015029	-2.511161	H2ac4/H2bc3/H3c4/H4c4/H3c2/H3c11/H4c18/H3c3/H2ac7/H2ac13/H2ac10/H4c1/H4c3
REACTOME_PRE_NOTCH_EXPRESSION_AND_PROCESSING	0.00015029	-2.511161	H2ac4/H2bc3/H3c4/H4c4/H3c2/H3c11/H4c18/H3c3/H2ac7/H2ac13/H2ac10/H4c1/H4c3
REACTOME_RHO_GTPASES_ACTIVATE_PKNS	0.00015029	-2.511161	H2ac4/H2bc3/H3c4/H4c4/H3c2/H3c11/H4c18/H3c3/H2ac7/H2ac13/H2ac10/H4c1/H4c3
REACTOME_RNA_POLYMERASE_I_PROMOTER_ESCAPE	0.00015029	-2.511161	H2ac4/H2bc3/H3c4/H4c4/H3c2/H3c11/H4c18/H3c3/H2ac7/H2ac13/H2ac10/H4c1/H4c3
REACTOME_RNA_POLYMERASE_I_TRANSCRIPTION	0.00015029	-2.511161	H2ac4/H2bc3/H3c4/H4c4/H3c2/H3c11/H4c18/H3c3/H2ac7/H2ac13/H2ac10/H4c1/H4c3
REACTOME_SIRT1_NEGATIVELY_REGULATES_RRNA_EXPRESSION	0.00015029	-2.511161	H2ac4/H2bc3/H3c4/H4c4/H3c2/H3c11/H4c18/H3c3/H2ac7/H2ac13/H2ac10/H4c1/H4c3
REACTOME_TRANSCRIPTIONAL_REGULATION_BY_SMALL_RNAS	0.00015029	-2.511161	H2ac4/H2bc3/H3c4/H4c4/H3c2/H3c11/H4c18/H3c3/H2ac7/H2ac13/H2ac10/H4c1/H4c3
REACTOME_INTERFERON_GAMMA_SIGNALING	0.00015029	2.26109789	Oas1g/Irf7/Trim34a/Gbp2/Gbp3/Gbp9/Oas2/Oas3/Oas1a/Gbp7/Sp100/H2-T23/Irf9/Trim12c/Trim21
REACTOME_MEIOTIC_RECOMBINATION	0.00016872	-2.4358582	H4c6/H2ac4/H2bc3/H3c4/H4c4/H3c2/H3c11/H4c18/H3c3/H2ac7/H2ac13/H2ac10/H4c1/H4c3
REACTOME_MEIOSIS	0.00021987	-2.4255436	Msh5/H4c6/H2ac4/Terf2ip/H2bc3/H3c4/H4c4/H3c2/H3c11/H4c18/H3c3/H2ac7/H2ac13/H2ac10/H4c1/H4c3
REACTOME_REPRODUCTION	0.00021987	-2.4255436	Msh5/H4c6/H2ac4/Terf2ip/H2bc3/H3c4/H4c4/H3c2/H3c11/H4c18/H3c3/H2ac7/H2ac13/H2ac10/H4c1/H4c3
REACTOME_ACTIVATION_OF_ANTERIOR_HOX_GENES_IN_HINDBRAIN	0.00027139	-2.4234426	H4c6/H2ac4/H2bc3/H3c4/H4c4/H3c2/H3c11/H4c18/H3c3/H2ac7/H2ac13/H2ac10/H4c1/H4c3/Pcgf2
REACTOME_RUNX1_REGULATES_GENES_INVOLVED_IN_MEGAKARYC	0.00035311	-2.3855286	H4c6/H2ac4/H2bc3/H3c4/H4c4/H3c2/H3c11/Prkcq/H4c18/H3c3/H2ac7/H2ac13/H2ac10/H4c1/H4c3
REACTOME_SENESCENCE_ASSOCIATED_SECRETORY_PHENOTYPE_SAS	0.00037369	-2.3706119	Ube2c/H4c6/H2ac4/Ube2s/H2bc3/H3c4/H4c4/H3c2/H3c11/H4c18/H3c3/H2ac7/H2ac13/H2ac10/H4c1/H4c3
REACTOME_ESTROGEN_DEPENDENT_GENE_EXPRESSION	0.00051687	-2.3231786	H4c6/H2ac4/H2bc3/H3c4/H4c4/H3c2/H3c11/H4c18/H3c3/H2ac7/H2ac13/H2ac10/H4c1/H4c3
REACTOME_TRANSCRIPTIONAL_REGULATION_OF_GRANULOPOIESIS	0.00051687	-2.3231786	H4c6/H2ac4/H2bc3/H3c4/H4c4/H3c2/H3c11/H4c18/H3c3/H2ac7/H2ac13/H2ac10/H4c1/H4c3
REACTOME_BASE_EXCISION_REPAIR	0.00094125	-2.2740972	H2ac4/Terf2ip/H2bc3/H4c4/H4c18/H2ac7/H2ac13/H2ac10/H4c1/H4c3
REACTOME_BASE_EXCISION_REPAIR_AP_SITE_FORMATION	0.00094125	-2.2740972	H2ac4/Terf2ip/H2bc3/H4c4/H4c18/H2ac7/H2ac13/H2ac10/H4c1/H4c3
REACTOME_INHIBITION_OF_DNA_RECOMBINATION_AT_TELOMERE	0.00094125	-2.2740972	H2ac4/Terf2ip/H2bc3/H4c4/H4c18/H2ac7/H2ac13/H2ac10/H4c1/H4c3
REACTOME_RECOGNITION_AND_ASSOCIATION_OF_DNA_GLYCOSYLA	0.00094125	-2.2740972	H2ac4/Terf2ip/H2bc3/H4c4/H4c18/H2ac7/H2ac13/H2ac10/H4c1/H4c3
REACTOME_DDX58_IFIH1_MEDIATED_INDUCTION_OF_INTERFERON_	0.00114836	2.03824808	Irf7/Rigi/Isg15/Dhx58/Ifih1/Uba7/Ube2l6
REACTOME_DEPOSITION_OF_NEW_CENPA_CONTAINING_NUCLEOSC	0.00155639	-2.2199935	H2ac4/H2bc3/H4c4/H4c18/H2ac7/H2ac13/H2ac10/H4c1/H4c3
REACTOME_INNATE_IMMUNE_SYSTEM	0.00166618	1.90444537	Plac8/Irf7/Tlr4/Rigi/Isg15/Dhx58/Fcer1a/Lat/Lgals3/Adgrg3/Cd53/H2-T23/F2/Casp4/Ifih1/Nod1/Tec/Trim21/Uba7/Ube2l6/Psmb9/Tlr6/Colec11/Lyz2/Cd55/Muc13/Fyn/Aldoc/Bst2/H2-Q10
REACTOME_MITOTIC_PROPHASE	0.00200645	-2.1754593	Banf1/H4c6/H2ac4/H2bc3/H3c4/H4c4/H3c2/H3c11/H4c18/H3c3/H2ac7/H2ac13/H2ac10/H4c1/H4c3
REACTOME_MEIOTIC_SYNAPSIS	0.00313021	-2.1421661	H4c6/H2ac4/Terf2ip/H2bc3/H4c4/H4c18/H2ac7/H2ac13/H2ac10/H4c1/H4c3
REACTOME_DNA_REPLICATION	0.00682982	-1.9905663	Ube2c/H4c6/H2ac4/Ube2s/H2bc3/H3c4/H4c4/H3c2/H3c11/H4c18/H3c3/H2ac7/H2ac13/H2ac10/H4c1/H4c3
REACTOME_DNA_DAMAGE_TELOMERE_STRESS_INDUCED_SENESCEN	0.00770254	-2.0012128	H4c6/H2ac4/Terf2ip/H2bc3/H4c4/H4c18/H2ac7/H2ac13/H2ac10/H4c1/H4c3
REACTOME_TCF_DEPENDENT_SIGNALING_IN_RESPONSE_TO_WNT	0.01032481	-1.9522047	H4c6/H2ac4/H2bc3/Tle1/H3c4/H4c4/H3c2/H3c11/H4c18/H3c3/H2ac7/H2ac13/H2ac10/H4c1/H4c3/Hecw1
REACTOME_CHROMOSOME_MAINTENANCE	0.01053234	-1.9677204	H4c6/H2ac4/Terf2ip/H2bc3/H4c4/H4c18/H2ac7/H2ac13/H2ac10/H4c1/H4c3
REACTOME_TELOMERE_MAINTENANCE	0.01053234	-1.9677204	H4c6/H2ac4/Terf2ip/H2bc3/H4c4/H4c18/H2ac7/H2ac13/H2ac10/H4c1/H4c3
REACTOME_DNA_REPLICATION_PRE_INITIATION	0.01898907	-1.8330608	H4c6/H2ac4/H2bc3/H3c4/H4c4/H3c2/H3c11/H4c18/H3c3/H2ac7/H2ac13/H2ac10/H4c1/H4c3
REACTOME_PKMTS_METHYLATE_HISTONE_LYSINES	0.0207272	-1.8730125	H4c6/H3c4/H4c4/H3c2/H3c11/H4c18/H3c3/H4c1/H4c3
REACTOME_SIGNALING_BY_WNT	0.02658674	-1.7617112	H4c6/H2ac4/H2bc3/Tle1/H3c4/H4c4/H3c2/H3c11/H4c18/H3c3/H2ac7/H2ac13/H2ac10/H4c1/H4c3/Hecw1
REACTOME_CLASS_I_MHC_MEDIATED_ANTIGEN_PROCESSING_PRES	0.03208192	1.66598965	Tlr4/Rnf213/H2-T23/Zbtb16/Trim21/Herc6/Uba7/Ube2l6/Psmb9/Tlr6/Tap1/Tapbp/H2-Q10/Erap1/Psmb8/Prkn/Dtx3l/Ncf1/B2m/Rnf114/H2-M3/H2-Q1/S100a1/Gm8909/Psme1
REACTOME_METABOLISM_OF_RNA	0.03992251	-1.6623736	Cpsf1/Lsm5/Ctu2/Lcmt2/Snrpd3/Rpl22l1/Snrpf/Rpl34/Utp3/Rbm17/Lsm7/Las1l/Cnot2/Rps16/Ncl/Zfp36/Igf2bp3
REACTOME_RUNX1_REGULATES_TRANSCRIPTION_OF_GENES_INVOL	0.04299585	-1.7418982	H4c6/H2ac4/H2bc3/H3c4/H4c4/H3c2/H3c11/H4c18/H3c3/H2ac7/H2ac13/H2ac10/H4c1/H4c3

IDDay2	p.adjust	NES	core_enrichment
HALLMARK_INTERFERON_GAMMA_RESPONSE	4.0445E-08	3.04674057	Fcgr1/Isg15/Rsad2/Usp18/Casp4/Gbp3/Txnip/Ddx60/Tnfsf10/Gbp9/Rtp4/Mthfd2/H2-Eb1/Epsti1/Xaf1/Cd69/Lgals3bp/Rigi/Gch1/Mx2/Pim1/Socs1/St8sia4/Btg1/B2m/Lcp2/Psmb8/Oas2/Oasl1/Cmpk2/Ifi27/Ifit1bl1/Samd9l
HALLMARK_INTERFERON_ALPHA_RESPONSE	9.3653E-06	2.80048022	Oas1g/Isg15/Rsad2/Usp18/Gbp3/Txnip/Ddx60/Rtp4/Epsti1/Lgals3bp/Mx2/Oas1a/B2m/Trim12c/Psmb8/Oasl1/Cmpk2/Tmem140/Ifi27/Samd9l
HALLMARK_APOPTOSIS	0.00078245	2.42371109	Atf3/Jun/Ddit3/Cth/Casp4/Txnip/Tnfsf10/F2r/Ank/Cd69/Gch1/Gadd45a/Lgals3/Pmaip1/H1f0
HALLMARK_KRAS_SIGNALING_UP	0.00487177	2.1661723	Etv4/Btc/Atg10/Fgf9/Ank/Dusp6/Hkdc1/Ano1/Lcp1/Kif5c/Cxcr4/Zfp639/Psmb8/Cbr4
HALLMARK_P53_PATHWAY	0.00487177	2.07514652	Trib3/Atf3/Jun/Slc7a11/Ddit3/Nupr1/Txnip/F2r/Coq8a/Slc3a2/Nol8/Socs1/Btg1/Gadd45a
HALLMARK_MTORC1_SIGNALING	0.00487177	2.047237	Trib3/Slc7a11/Ddit3/Cth/Nupr1/Niban1/Mthfd2/Add3/Slc1a4/Dapp1/Cxcr4/Hspa9/Psph/Ifrd1/Got1/Eprs/Ctsc/Psat1/Eef1e1/Nufip1/Asns/Nfil3/Ppp1r15a/Wars1/Etf1
HALLMARK_UNFOLDED_PROTEIN_RESPONSE	0.00581784	2.11276783	Atf3/Chac1/Stc2/Cebpg/Mthfd2/Slc1a4/Herpud1/Hspa9/Aldh18a1/Tars/Ifit1bl1/Atf4/Cebpb/Psat1/Eif4ebp1/Asns/Nop14/Exosc1/Sdad1/Npm1/Nop56/Xpot/Dkc1
HALLMARK_MYC_TARGETS_V1	0.00909219	2.03913594	Ifrd1/Eprs/Gnl3/Cnbp/Orc2/Rsl1d1/Pwp1/Etf1/Apex1/Eif3j2/Npm1/Ddx21/Nop56/Xpot/Tufm/Srm/Clns1a/Syncrip/Hspd1/Dhx15/Eif2s2/Usp1/Set/Cstf2/Serbp1/Nolc1
HALLMARK_MITOTIC_SPINDLE	0.01488213	-1.9371547	Arhgap4/Arhgef12/Rhof/Nf1/Map3k11/Clasp1/Wasf2/Kif3c/Tiam1/Cdc42ep2/Tuba4a/Wasl/Abl1/Arhgef7/Pif1/Arl8a/Cep250/Notch2/Arap3/Ssh2/Taok2/Prex1/Map1s/Abr/Arhgdia/Akap13/Wasf1/Espl1/Plk1/Arhgef11/Bcar1/Mark4/Plekhg2/Mid1
HALLMARK_ALLOGRAFT_REJECTION	0.02758873	1.86018514	Ccr1/Thy1/F2r/Itk/Rars/Il4/H2-Oa/Socs1/St8sia4/B2m/Tlr6/Lcp2/Lck/Irf7/Spi1/Aars/Cd1d1/Wars1/Ptprc/Eif3j2/Hif1a/Dars
KEGG_SYSTEMIC_LUPUS_ERYTHEMATOSUS	0.00018639	2.73473735	Fcgr1/H3c8/H2-Eb1/H3f3b/H4c16/H4c14/H4c11/H3c7/H2-Oa/H3c1/H3c11/H3c10/H4c12/H3c4/H2ac20/H4c1/H4c18/H3c2/H2ac6/H4c2/H4c8/H2ac8/H2bc8/H4c6/H2ac4/H3c3/H2ac13/Fcgr2b/H4c4/H2ac12
KEGG_AMINOACYL_TRNA_BIOSYNTHESIS	0.00196545	2.48467987	Rars/Nars/Lars/Sepsecs/Tars/Eprs/Gars/Sars/Rars2/Aars/Wars1/Ears2/Dars
KEGG_LEISHMANIA_INFECTION	0.01019058	2.35370727	Tlr4/Fcgr1/Jun/H2-Eb1/Il4/H2-Oa
REACTOME_RNA_POLYMERASE_I_PROMOTER_ESCAPE	5.5601E-06	3.00382313	H3c8/H3f3b/H4c16/H4c14/H4c11/H3c7/Polr1f/H3c1/H3c11/H3c10/H4c12/H3c4/H2ac20/H4c1/Taf1b/H4c18/H3c2/H4c2/H4c8/H2bc8/Gtf2h1/H4c6/Polr2h/H2ac4/H3c3
REACTOME_ASSEMBLY_OF_THE_ORC_COMPLEX_AT_THE_ORIGIN_OF_REPLICATION	1.2632E-05	2.94715161	H3c8/H3f3b/H4c16/H4c14/H4c11/H3c7/H3c1/H3c11/H3c10/H4c12/H3c4/H2ac20/H4c1/Orc5/Orc2/H4c18/H3c2/H4c2/H4c8/H2bc8/H4c6/Orc4/H2ac4/H3c3
REACTOME_SIRT1_NEGATIVELY_REGULATES_RRNA_EXPRESSION	1.2632E-05	2.90302652	H3c8/H3f3b/H4c16/H4c14/H4c11/H3c7/H3c1/H3c11/H3c10/H4c12/H3c4/H2ac20/H4c1/Taf1b/H4c18/H3c2/Sirt1/H4c2/H4c8/H2bc8/H4c6/H2ac4/H3c3
REACTOME_DNA_METHYLATION	1.5816E-05	2.86494745	H3c8/H3f3b/H4c16/H4c14/H4c11/H3c7/H3c1/H3c11/H3c10/H4c12/H3c4/H2ac20/H4c1/H4c18/H3c2/H4c2/H4c8/H2bc8/H4c6/H2ac4/H3c3
REACTOME_MEIOTIC_RECOMBINATION	2.336E-05	2.83840378	H3c8/H3f3b/H4c16/H4c14/H4c11/H3c7/H3c1/H3c11/H3c10/H4c12/H3c4/H2ac20/H4c1/H4c18/H3c2/H4c2/H4c8/H2bc8/H4c6/H2ac4/H3c3/Rad51c/H2ac13/Nbn/H4c4
REACTOME_DNA_REPLICATION_PRE_INITIATION	2.3475E-05	2.75135054	H3c8/H3f3b/H4c16/H4c14/H4c11/H3c7/H3c1/H3c11/H3c10/Psmb8/H4c12/H3c4/H2ac20/H4c1/Orc5/Orc2/H4c18/H3c2/H4c2/H4c8/H2bc8/H4c6/Orc4/H2ac4/H3c3/Psmb9/H2ac13
REACTOME_ACTIVATED_PKN1_STIMULATES_TRANSCRIPTION_OF_AR_ANDROGEN_R	4.7669E-05	2.77979503	H3c8/H3f3b/H4c16/H4c14/H4c11/H3c7/H3c1/H3c11/H3c10/H4c12/H3c4/H2ac20/H4c1/H4c18/H3c2/H4c2/H4c8/H2bc8/H4c6/H2ac4/H3c3
REACTOME_RNA_POLYMERASE_I_TRANSCRIPTION	4.7669E-05	2.66779585	H3c8/H3f3b/H4c16/H4c14/H4c11/H3c7/Polr1f/H3c1/H3c11/H3c10/H4c12/H3c4/H2ac20/H4c1/Taf1b/H4c18/H3c2/H4c2/H4c8/H2bc8/Gtf2h1/H4c6/Polr2h/H2ac4/H3c3
REACTOME_NEGATIVE_EPIGENETIC_REGULATION_OF_RRNA_EXPRESSION	6.4264E-05	2.63613808	H3c8/H3f3b/H4c16/H4c14/H4c11/H3c7/Polr1f/H3c1/H3c11/H3c10/H4c12/H3c4/H2ac20/H4c1/Taf1b/H4c18/H3c2/Sirt1/H4c2/H4c8/H2bc8/Gtf2h1/H4c6/Polr2h/H2ac4/H3c3
REACTOME_CYTOKINE_SIGNALING_IN_IMMUNE_SYSTEM	7.7516E-05	2.28952089	Ccr1/Oas1g/Fcgr1/Havcr2/Jun/Trim34a/Isg15/Rsad2/Usp18/Gbp3/Gbp9/H3c8/Il1rl1/Dusp6/H2-Eb1/Il6ra/Xaf1/Il21r/Car1/Osm/Il4/Gbp7/Rigi/Lcp1/Mx2/H3c7/Oas1a/Pim1/Socs1/Hspa9/H3c1/H3c11/H3c10/B2m/Trim12c/Psmb8/Oas2/Oasl1/Tnfrsf13b/H3c4/Egr1
REACTOME_B_WICH_COMPLEX_POSITIVELY_REGULATES_RRNA_EXPRESSION	0.0001218	2.67849148	H3c8/H3f3b/H4c16/H4c14/H4c11/H3c7/Polr1f/H3c1/H3c11/H3c10/H4c12/H3c4/H2ac20/H4c1/Taf1b/H4c18/H3c2/H4c2/H4c8/H2bc8/H4c6/Ddx21/Polr2h/H2ac4/H3c3
REACTOME_PRC2_METHYLATES_HISTONES_AND_DNA	0.00017161	2.67341669	H3c8/H3f3b/H4c16/H4c14/H4c11/H3c7/H3c1/H3c11/H3c10/H4c12/H3c4/H2ac20/H4c1/H4c18/H3c2/H4c2/H4c8/H2bc8/H4c6/H2ac4/H3c3/Aebp2/H2ac13
REACTOME_INTERFERON_ALPHA_BETA_SIGNALING	0.00019514	2.70860325	Oas1g/Isg15/Rsad2/Usp18/Xaf1/Mx2/Oas1a/Socs1/Psmb8/Oas2/Oasl1/Egr1/Ifi27/Ifit1bl1
REACTOME_METABOLISM_OF_AMINO_ACIDS_AND_DERIVATIVES	0.00019514	2.52957304	Cth/Rimklb/Aspa/Gpt2/Rars/Kmo/Paox/Slc3a2/Kyat1/Psph/Got1/Aldh18a1/Psmb8/Lars/Pycr1/Sepsecs/Serinc1/Eprs/Lipt1/Sars/Dbt/Psat1/Eef1e1/Rpl22l1/Asns/Lipt2/Azin1/Mtap/Hoga1/Dars
REACTOME_SENESCENCE_ASSOCIATED_SECRETORY_PHENOTYPE_SASP	0.00019514	2.51139931	Jun/H3c8/H3f3b/H4c16/H4c14/H4c11/H3c7/H3c1/H3c11/H3c10/H4c12/H3c4/H2ac20/H4c1/H4c18/H3c2/Cebpb/H4c2/H4c8/H2bc8/H4c6/H2ac4/H3c3
REACTOME_ERCC6_CSB_AND_EHMT2_G9A_POSITIVELY_REGULATE_RRNA_EXPRESS	0.00022631	2.54999513	H3c8/H3f3b/H4c16/H4c14/H4c11/H3c7/H3c1/H3c11/H3c10/H4c12/H3c4/H2ac20/H4c1/H4c18/H3c2/H4c2/H4c8/H2bc8/H4c6/H2ac4/H3c3
REACTOME_DNA_REPLICATION	0.00022631	2.51755021	H3c8/H3f3b/H4c16/H4c14/H4c11/H3c7/H3c1/H3c11/H3c10/Psmb8/H4c12/H3c4/H2ac20/H4c1/Orc5/Orc2/H4c18/H3c2/H4c2/H4c8/H2bc8/H4c6/Orc4/H2ac4/H3c3/Psmb9/H2ac13/H4c4/Anapc16/Orc6
REACTOME_MEIOSIS	0.00022631	2.47616985	H3c8/H3f3b/H4c16/H4c14/H4c11/H3c7/H3c1/H3c11/H3c10/H4c12/H3c4/Stag2/H2ac20/H4c1/H4c18/H3c2/H4c2/H4c8/H2bc8/H4c6/H2ac4/H3c3/Rad51c/H2ac13/Nbn/H4c4
REACTOME_CONDENSATION_OF_PROPHASE_CHROMOSOMES	0.0002923	2.51706347	H3c8/H3f3b/H4c16/H4c14/H4c11/H3c7/H3c1/H3c11/H3c10/H4c12/H3c4/H2ac20/H4c1/H4c18/H3c2/H4c2/H4c8/H2bc8/H4c6/H2ac4/H3c3
REACTOME_RRNA_PROCESSING	0.0002923	2.50269308	Rpp40/Wdr43/Thumpd1/Nop58/Mphosph10/Ltv1/Rpp38/Wdr75/Tsr1/Gnl3/Trmt10c/Rpl22l1/Wdr12/Wdr36/Krr1/Nop14/Exosc1/Utp15/Rpp14/Nip7/Dimt1/Rcl1/Riok2/Ddx21/Nop56/Nol11/Tfb1m/Dkc1/Mrm3/Prorp/Xrn2/Pwp2/Gar1/Imp4/Utp18/Pno1/Riok1
REACTOME_TRANSLATION	0.00033028	2.5571895	Rars/Nars/Lars/Tars/Eprs/Gars/Sars/Eef1e1/Rpl22l1/Rars2/Eif4ebp1/Aars/Wars1/Etf1/Mrrf/Eif3j2/Ears2/Dars/Mrpl19/N6amt1/Mrpl15/Eif2b3/Farsb/Tufm/Eif2s2/Mrpl32/Eif3c/Mrpl16/Yars/Mrps31
REACTOME_TRNA_AMINOACYLATION	0.00034303	2.544999	Rars/Nars/Lars/Tars/Eprs/Gars/Sars/Eef1e1/Rars2/Aars/Wars1/Ears2/Dars
REACTOME_POSITIVE_EPIGENETIC_REGULATION_OF_RRNA_EXPRESSION	0.00034303	2.47457057	H3c8/H3f3b/H4c16/H4c14/H4c11/H3c7/Polr1f/H3c1/H3c11/H3c10/H4c12/H3c4/H2ac20/H4c1/Taf1b/H4c18/H3c2/H4c2/H4c8/H2bc8/H4c6/Ddx21/Polr2h/H2ac4/H3c3
REACTOME_REPRODUCTION	0.00059171	2.41813913	H3c8/H3f3b/H4c16/H4c14/H4c11/Izumo1/H3c7/H3c1/H3c11/H3c10/H4c12/H3c4/Stag2/H2ac20/H4c1/H4c18/H3c2/H4c2/H4c8/H2bc8/H4c6/H2ac4/H3c3/Rad51c/H2ac13/Nbn/H4c4
REACTOME_RHO_GTPASES_ACTIVATE_PKNS	0.00066716	2.39818451	H3c8/H3f3b/H4c16/H4c14/H4c11/H3c7/H3c1/H3c11/H3c10/H4c12/H3c4/H2ac20/H4c1/H4c18/H3c2/H4c2/H4c8/H2bc8/H4c6/H2ac4/H3c3
REACTOME_RUNX1_REGULATES_TRANSCRIPTION_OF_GENES_INVOLVED_IN_DIFFER	0.00093079	2.44654837	Myb/H3c8/H3f3b/H4c16/H4c14/H4c11/H3c7/H3c1/H3c11/H3c10/Psmb8/H4c12/H3c4/Cbfb/H2ac20/H4c1/H4c18/H3c2/Spi1/H4c2/H4c8/H2bc8/H4c6/H2ac4/H3c3/Psmb9/H2ac13/Lmo2/H4c4
REACTOME_CDC42_GTPASE_CYCLE	0.0012264	-2.2265123	Arhgap4/Arhgef12/Arhgdig/Tfrc/Map3k11/Jup/Srgap2/Tiam1/Cdc42ep2/Wasl/Arhgap21/Vav2/Plekhg3/Arhgef7/Iqgap3/Baiap2/Vangl1/Wdr81/Dnmbp/Steap3/Arhgap39/Git2/Arap3/Spata13/Arhgap35/Scrib/Prex1/Srgap3/Pak6/Arhgef19/Sh3pxd2a/Abr/Arhgdia/Wipf2/Pak4/Arhgap17/Git1/Arhgef11/Dock6/Plekhg2/Arhgap20/Arhgap33
REACTOME_RHO_GTPASE_CYCLE	0.0013864	-2.0330733	Pkn1/Efhd2/Pik3r2/Cavin1/Cpsf7/Zfp512b/Arhgap4/Arhgef12/Frs2/Rac3/Rhof/Arhgdig/Vangl2/Tfrc/Senp1/Wdr6/Map3k11/Jup/Srgap2/Nisch/Csk/Wasf2/Tiam1/Cdc42ep2/Rapgef1/Sh3bp1/Wasl/Elmo2/Arhgap21/Vav2/Cyfip2/Nox1/Plekhg3/Arhgef7/Iqgap3/Baiap2/Vangl1/Kif14/Ptk2b/Diaph1/Wdr81/Dnmbp/Steap3/Actn1/Arhgap39/Git2/Rhpn1/Arap3/Spata13/Arhgap35/Plxnd1/Scrib/Prex1/Srgap3/Pak6/Arhgef19/Arhgef1/Sh3pxd2a/Abr/Taok3/Peak1/Arhgdia/Akap13/Wasf1/Wipf2/Pak4/Srrm1/Arhgap17/Whamm/Git1/Plekhg5/Prag1/Arhgef11/Plxna1/Dock6/Plekhg2/Spen/Arhgap20/Cit/Arhgap23/Arhgap33
REACTOME_RAC1_GTPASE_CYCLE	0.00161488	-2.1360885	Srgap2/Nisch/Wasf2/Tiam1/Sh3bp1/Wasl/Arhgap21/Vav2/Cyfip2/Nox1/Plekhg3/Arhgef7/Iqgap3/Baiap2/Vangl1/Arhgap39/Git2/Arap3/Spata13/Arhgap35/Prex1/Srgap3/Pak6/Arhgef19/Abr/Taok3/Arhgdia/Wasf1/Wipf2/Pak4/Arhgap17/Git1/Arhgef11/Dock6/Plekhg2/Arhgap20/Cit/Arhgap23/Arhgap33
REACTOME_DEPOSITION_OF_NEW_CENPA_CONTAINING_NUCLEOSOMES_AT_THE_C	0.00167662	2.3719158	H4c16/H4c14/H4c11/H4c12/H2ac20/H4c1/H4c18/H4c2/H4c8/H2bc8/Itgb3bp/H4c6/Npm1/H2ac4/H2ac13/Cenpq/H4c4
REACTOME_INTERFERON_SIGNALING	0.00167662	2.29772123	Oas1g/Fcgr1/Trim34a/Isg15/Rsad2/Usp18/Gbp3/Gbp9/H2-Eb1/Xaf1/Gbp7/Rigi/Mx2/Oas1a/Socs1/B2m/Trim12c/Psmb8/Oas2/Oasl1/Egr1/Ifi27/Ifit1bl1
REACTOME_RESPONSE_OF_EIF2AK1_HRI_TO_HEME_DEFICIENCY	0.00197799	2.3679339	Trib3/Atf3/Chac1/Ddit3/Cebpg/Atf4/Cebpb/Asns/Ppp1r15a
REACTOME_TRANSCRIPTIONAL_REGULATION_OF_GRANULOPOIESIS	0.00197799	2.27114817	Myb/H3c8/H3f3b/H4c16/Il6ra/H4c14/H4c11/H3c7/H3c1/H3c11/H3c10/H4c12/H3c4/Cbfb/H2ac20/H4c1/H4c18/H3c2/Cebpb/Spi1/H4c2/H4c8/Creb1/H2bc8/H4c6/H2ac4/H3c3
REACTOME_DISEASES_OF_PROGRAMMED_CELL_DEATH	0.00251034	2.18360095	Jun/H3c8/H3f3b/H4c16/H4c14/H4c11/H3c7/H3c1/H3c11/H3c10/H4c12/H3c4/H2ac20/H4c1/H4c18/H3c2
REACTOME_CYTOSOLIC_TRNA_AMINOACYLATION	0.0025398	2.35086488	Rars/Nars/Lars/Tars/Eprs/Gars/Sars/Eef1e1/Aars/Wars1/Dars
REACTOME_NERVOUS_SYSTEM_DEVELOPMENT	0.0025398	-2.0101639	Nrtn/Pik3r2/Map2k2/Ubc/Lamc1/Ephb6/Msn/Dok4/Arhgef12/Frs2/Dpysl2/Ncstn/Pabpc1/Utrn/Clasp1/Sptbn4/Srgap2/Efna3/Rap1gap/Tiam1/Tubb4b/Prkaca/Adgrv1/Cacnb1/Tuba4a/Wasl/Pdlim7/Vav2/Abl1/Arhgef7/Hspa8/Tuba1c/Smarca4/Arhgap39/Pip5k1c/Ap2a1/Arhgap35/Ephb3/Srebf2/Plxnd1/Cacnb3/Drp2/Agrn/Scn10a/Srgap3/Dag1/Pak6/Tubb1/Zswim8/Pak4/Efnb3/Cacna1c/Sptb/Gpc1/Itga10/Git1/Dlg4/L1cam/Cacna1h/Arhgef11/Sema4d/Tubb4a/Plxna1/Msi1/Dnm1/Col4a3/Ank1/Cacna1g/Met/Ret
REACTOME_AMYLOID_FIBER_FORMATION	0.0026966	2.2463957	Lyz2/H3c8/H3f3b/H4c16/H4c14/H4c11/H3c7/H3c1/H3c11/H3c10/B2m/H4c12/H3c4/H2ac20/H4c1/H4c18/H3c2/H4c2/H4c8/H2bc8/Tspan14/H4c6/H2ac4/H3c3/Siah1a/H2ac13
REACTOME_RHOQ_GTPASE_CYCLE	0.0040601	-2.2652479	Tfrc/Jup/Srgap2/Cdc42ep2/Wasl/Arhgap21/Plekhg3/Arhgef7/Iqgap3/Vangl1/Steap3/Git2/Arhgap35/Scrib/Prex1/Pak4/Arhgap17/Git1/Arhgap33
REACTOME_RESPONSE_OF_EIF2AK4_GCN2_TO_AMINO_ACID_DEFICIENCY	0.00583645	2.24391428	Trib3/Atf3/Ddit3/Cebpg/Atf4/Cebpb/Rpl22l1/Asns/Impact
REACTOME_HCMV_LATE_EVENTS	0.00616134	2.08135379	H3c8/H4c16/H4c14/H4c11/H3c7/H3c1/H3c11/H3c10/H4c12/H3c4/H2ac20/H4c1/H4c18/H3c2/H2ac6/Nup43/H4c2/H4c8/H2ac8/H2bc8/H4c6/H2ac4/H3c3/H2ac13/H4c4/H2ac12/Nupl2
REACTOME_TRNA_MODIFICATION_IN_THE_NUCLEUS_AND_CYTOSOL	0.00666881	2.24903227	Qtrt2/Trmt61a/Eprs/Mettl1/Trdmt1/Trmt10a/Trp53rka/Trmt6/Pus3/Nsun2/Tyw5/Tyw1/Qtrt1/Alkbh8/Trmt11
REACTOME_RHOA_GTPASE_CYCLE	0.0070321	-2.1201535	Pkn1/Pik3r2/Cavin1/Arhgap4/Arhgef12/Tfrc/Jup/Tiam1/Arhgap21/Vav2/Plekhg3/Arhgef7/Iqgap3/Vangl1/Diaph1/Arhgap39/Rhpn1/Arap3/Arhgap35/Prex1/Arhgef19/Arhgef1/Abr/Arhgdia/Akap13/Plekhg5/Arhgef11/Arhgap20/Cit/Arhgap23
REACTOME_BASE_EXCISION_REPAIR_AP_SITE_FORMATION	0.00754992	2.13415526	H4c16/H4c14/H4c11/H4c12/H2ac20/H4c1/H4c18/H4c2/H4c8/H2bc8/H4c6/H2ac4/H2ac13/H4c4
REACTOME_RECOGNITION_AND_ASSOCIATION_OF_DNA_GLYCOSYLASE_WITH_SITE_	0.00754992	2.13415526	H4c16/H4c14/H4c11/H4c12/H2ac20/H4c1/H4c18/H4c2/H4c8/H2bc8/H4c6/H2ac4/H2ac13/H4c4
REACTOME_MITOCHONDRIAL_PROTEIN_IMPORT	0.007674	2.15461007	Hspa9/Timm8a1/Chchd5/Chchd4/Coa4/Hscb/Timm10/Timm21/Timm9/Hspd1
REACTOME_TRANSCRIPTIONAL_REGULATION_BY_SMALL_RNAS	0.007674	2.03111128	H3c8/H3f3b/H4c16/H4c14/H4c11/H3c7/H3c1/H3c11/H3c10/H4c12/H3c4/H2ac20/H4c1/H4c18/H3c2/Nup43/H4c2/H4c8/H2bc8/H4c6/Polr2h/H2ac4/H3c3
REACTOME_NEURONAL_SYSTEM	0.007674	-2.0122443	Dlgap4/Rab3a/Tubb4b/Prkaca/Ppfia3/Epb41l1/Cacnb1/Tuba4a/Ptprs/Stxbp1/Arhgef7/Hspa8/Camk2g/Nrgn/Tuba1c/Shank1/Hcn2/Ap2a1/Cacnb3/Ppfia1/Dlg2/Tubb1/Git1/Dlg4/Vamp2/Kcnd1/Tubb4a/Gabbr1/Kcnmb4/Nlgn3/Grik5/Ache/Gabrq/Nlgn2/Tspoap1
REACTOME_HDACS_DEACETYLATE_HISTONES	0.01184107	2.07766487	H3c8/H4c16/H4c14/H4c11/H3c7/H3c1/H3c11/H3c10/H4c12/H3c4/H2ac20/H4c1/H4c18/H3c2/H2ac6/H4c2/H4c8/H2ac8/H2bc8/H4c6/H2ac4/H3c3
REACTOME_ESR_MEDIATED_SIGNALING	0.01319813	1.93820759	Jun/Btc/Myb/H3c8/H3f3b/H4c16/H4c14/H4c11/H3c7/H3c1/H3c11/H3c10/Gng2/H4c12/H3c4/Stag2/Cbfb/H2ac20/H4c1/H4c18/H3c2/Egf/Gtf2f2/H4c2/H4c8/Creb1
REACTOME_TRNA_PROCESSING	0.01667917	2.03033816	Rpp40/Qtrt2/Rpp38/Trmt61a/Eprs/Trmt10c/Trnt1/Mettl1/Trdmt1/Trmt10a/Nup43/Trp53rka/Rpp14/Trmt6/Pus3/Nsun2/Tyw5/Xpot/Tsen15/Prorp/Nupl2/Tyw1/Qtrt1/Cstf2/Alkbh8/Nup54/Nupl1/Trmt11
REACTOME_ESTROGEN_DEPENDENT_GENE_EXPRESSION	0.01951315	1.92351736	Jun/Myb/H3c8/H3f3b/H4c16/H4c14/H4c11/H3c7/H3c1/H3c11/H3c10/H4c12/H3c4/Stag2/Cbfb/H2ac20/H4c1/H4c18/H3c2
REACTOME_RHOC_GTPASE_CYCLE	0.02384966	-2.0186014	Pkn1/Cavin1/Arhgef12/Tfrc/Jup/Arhgap21/Vav2/Iqgap3/Vangl1/Diaph1/Arhgap39/Arhgap35/Prex1/Arhgef1/Abr/Arhgdia/Akap13/Arhgef11/Cit
REACTOME_EPIGENETIC_REGULATION_OF_GENE_EXPRESSION	0.02403803	1.93555357	H3c8/H3f3b/H4c16/H4c14/H4c11/H3c7/Polr1f/H3c1/H3c11/H3c10/H4c12/H3c4/H2ac20/H4c1/Taf1b/H4c18/H3c2/Sirt1/H4c2/H4c8/H2bc8/Gtf2h1/H4c6/Ddx21/Polr2h/H2ac4/H3c3
REACTOME_SIGNALING_BY_INTERLEUKINS	0.02982412	1.72731275	Ccr1/Havcr2/Jun/H3c8/Il1rl1/Dusp6/Il6ra/Il21r/Car1/Osm/Il4/Lcp1/H3c7/Pim1/Socs1/Hspa9/H3c1/H3c11/H3c10/Psmb8
REACTOME_ATF4_ACTIVATES_GENES_IN_RESPONSE_TO_ENDOPLASMIC_RETICULUM	0.0303264	1.96752519	Atf3/Ddit3/Cebpg/Herpud1/Atf4/Cebpb/Asns/Exosc1
REACTOME_INTERFERON_GAMMA_SIGNALING	0.0303264	1.89558797	Oas1g/Fcgr1/Trim34a/Gbp3/Gbp9/H2-Eb1/Gbp7/Oas1a/Socs1/B2m/Trim12c/Oas2/Oasl1
REACTOME_HDMS_DEMETHYLATE_HISTONES	0.03321104	1.93158133	H3c8/H4c16/H4c14/H4c11/H3c7/H3c1/H3c11/H3c10/H4c12/H3c4/H4c1/H4c18/H3c2/H4c2/H4c8
REACTOME_PROCESSING_OF_DNA_DOUBLE_STRAND_BREAK_ENDS	0.03321104	1.88697196	H4c16/H4c14/H4c11/H4c12/Abraxas1/H4c1/H4c18/Rad1/H4c2/H4c8/H2bc8/H4c6/Rad17/Nbn/H4c4/Kat5/Chek1/Atr/Rbbp8/Ppp4r2/Herc2
REACTOME_MITOTIC_PROPHASE	0.03402743	1.87227674	H3c8/H3f3b/H4c16/H4c14/H4c11/H3c7/H3c1/H3c11/H3c10/H4c12/H3c4/H2ac20/H4c1/Nek6/H4c18/H3c2/Nup43/H4c2/H4c8/Blzf1/H2bc8/H4c6/H2ac4/H3c3
REACTOME_RRNA_MODIFICATION_IN_THE_NUCLEUS_AND_CYTOSOL	0.03465533	2.03741166	Wdr43/Thumpd1/Nop58/Mphosph10/Wdr75/Wdr36/Krr1/Nop14/Utp15/Dimt1/Rcl1/Nop56/Nol11/Dkc1/Pwp2/Gar1/Imp4/Utp18/Pno1/Nat10/Nol6
REACTOME_RUNX1_INTERACTS_WITH_CO_FACTORS_WHOSE_PRECISE_EFFECT_ON_	0.04108188	-1.9835223	Phc1/Arid1b/Smarcd3/Scmh1/Runx1/Cbx6/Smarcb1/Phc2/Smarca4/Smarcd1/Phc3/Smarcc2/Ep300/Hipk2/Arid1a
REACTOME_GENE_SILENCING_BY_RNA	0.04108188	1.77911065	H3c8/H3f3b/H4c16/H4c14/H4c11/H3c7/H3c1/H3c11/H3c10/H4c12/H3c4/H2ac20/H4c1/H4c18/H3c2/Nup43/H4c2/H4c8/H2bc8/H4c6/Polr2h/H2ac4/H3c3
REACTOME_PERK_REGULATES_GENE_EXPRESSION	0.04153287	1.9419441	Atf3/Ddit3/Cebpg/Herpud1/Atf4/Cebpb/Asns/Exosc1
REACTOME_PROTEIN_PROTEIN_INTERACTIONS_AT_SYNAPSES	0.04350483	-1.9629664	Dlgap4/Ppfia3/Epb41l1/Ptprs/Stxbp1/Shank1/Ppfia1/Dlg2/Dlg4/Nlgn3/Nlgn2
REACTOME_ANTIGEN_PROCESSING_CROSS_PRESENTATION	0.04508015	1.97511414	Tlr4/Fcgr1/B2m/Tlr6/Psmb8
REACTOME_RHOB_GTPASE_CYCLE	0.04667262	-1.9047102	Pkn1/Pik3r2/Cavin1/Arhgef12/Arhgdig/Tfrc/Jup/Arhgap21/Vav2/Iqgap3/Vangl1/Diaph1/Arhgap39/Arhgap35/Prex1/Arhgef1/Abr/Akap13/Arhgef11/Cit
REACTOME_VESICLE_MEDIATED_TRANSPORT	0.04667262	-1.626163	Sec16a/Kif13b/Ubc/Calr/Scarb1/Madd/Eps15l1/Ldlrap1/Trip11/Klc2/Gbf1/Akt1/Synj1/Sec24c/Sbf1/Tfrc/Myo1c/Sptbn4/Cbl/Gdi1/Kifc1/Kif5a/Pum1/Kif3c/Rab11b/Rab3a/Tubb4b/Tbc1d16/Sec24b/Kif21b/Tuba4a/Man1c1/Wasl/Rin3/Trappc9/Arrb1/Sec31a/Sec24a/Rab43/Mvb12b/Hspa8/Tuba1c/Rin1/Kifc2/Pip5k1c/Pla2g6/Apoe/Ap2a1/Tbc1d24/Rgp1/Cux1/Tubb1/Dennd4b/Kifc5b/Dennd1a/Tsc2/Gga1/Gjc2/Sptb/Ubqln2/Vps37c/Epn1/Ulk1/Gga3/Vamp2/Fcho1/Tubb4a/Kif18b/Hbb-bt/Ldlr/Dnm1/Rab3il1/Ank1/Hba-a1
REACTOME_METABOLISM_OF_RNA	0.04669444	1.61872175	Rpp40/Wdr43/Thumpd1/Nop58/Apobec1/Mphosph10/Psmb8/Ltv1/Mettl3/Qtrt2/Rpp38/Trmt61a/Wdr75/Tsr1/Eprs/Gnl3/Trmt10c/Trnt1/Mettl1/Trdmt1/Prmt5/Trmt10a/Rpl22l1/Gemin2/Gtf2f2/Wdr12/Nup43/Wdr36/Krr1/Nop14/Etf1/Trp53rka/Exosc1/Mettl14/Utp15/Rnmt/Rpp14/Trmt6/Gtf2h1/Pus3/Nip7/Dimt1/Rcl1/Slu7/Riok2/Ddx21/Polr2h/Nsun2/Nop56/Tyw5/Xpot/Nol11/Psmb9/Tfb1m/Dkc1/Clns1a/Tnpo1/Tsen15/Mrm3/Dhx15/Prorp/Phax/Nupl2/Pnrc2/Tyw1/Xrn2/Gemin6/Qtrt1/Set/Cstf2/Pwp2/Gar1/Alkbh8/Cpsf3/Nup54/Imp4/Utp18/Pno1/Riok1/Nupl1/Trmt11/Srsf10
REACTOME_NONHOMOLOGOUS_END_JOINING_NHEJ	0.04715922	1.93836832	H4c16/H4c14/H4c11/Xrcc5/H4c12/Abraxas1/H4c1/H4c18/H4c2/H4c8/H2bc8/H4c6/Tdp2/Nbn/H4c4/Kat5
REACTOME_BASE_EXCISION_REPAIR	0.04715922	1.93643389	H4c16/H4c14/H4c11/H4c12/H2ac20/H4c1/H4c18/H4c2/H4c8/Apex1/H2bc8/H4c6/H2ac4/H2ac13/H4c4
REACTOME_RAC2_GTPASE_CYCLE	0.04715922	-1.8884971	Tfrc/Wasf2/Tiam1/Arhgap21/Vav2/Vangl1/Arhgap39/Git2/Arhgap35/Prex1/Abr/Taok3/Arhgdia/Pak4/Arhgap17/Git1

IDDay5	p.adjust	NES	core_enrichment
HALLMARK_HEME_METABOLISM	2.3352E-09	-2.9937983	Dcaf11/Car2/Pigq/Tspo2/Narf/Ppp2r5b/E2f2/Fbxo9/Urod/Epb41/Eif2ak1/Nfe2/Dmtn/Foxo3/Hebp1/Snca/Prdx2/Top1/Mgst3/Btg2/Gypc/Map2k3/Smox/Slc22a4/Trak2/Ackr1/Alas2/Tal1/Sptb/Epb42/Hmbs/Klf1/Hbq1b/Slc30a10/Abcb6/Ermap/Marchf8/Klf3/Atp6v0a1/Cpox/Tent5c/Alad/Trim58/Tmcc2/Kel/Slc25a37/Trim10/Ucp2/Ank1/Rhd/Ranbp10/Hbb-bt/Slc4a1/Tfrc
HALLMARK_UNFOLDED_PROTEIN_RESPONSE	1.1052E-07	3.02802753	Chac1/Stc2/Herpud1/Hspa9/Eif4ebp1/Atf3/Mthfd2/Slc1a4/Aldh18a1/Psat1/Slc7a5/Cebpg/Cebpb/Ddit4/Asns/Sdad1/Exosc1/Atf4/Npm1/Ero1a/Dnajb9/Nop14/Tars/Xpot/Nop56
HALLMARK_ALLOGRAFT_REJECTION	0.00057322	2.36479529	Ccr1/F2r/Rars/Lck/Il12rb1/Ptprc/Thy1/Fcgr2b/Wars1/Tlr6/Npm1/Spi1/Lcp2/Csf1/Dars/Il4/B2m/Aars/Il4ra/Apbb1/Tap1/Itgal/Map4k1/Was
HALLMARK_MYC_TARGETS_V2	0.00057322	2.35827959	Wdr43/Gnl3/Srm/Npm1/Pus1/Mphosph10/Nip7/Dusp2/Prmt3/Nop56/Nop2/Grwd1/Nop16/Pes1/Rrp12/Exosc5/Myc/Wdr74/Nolc1/Rrp9/Rcl1/Mrto4/Hspd1
HALLMARK_MYC_TARGETS_V1	0.00115145	2.16178835	Eprs/Apex1/Gnl3/Srm/Npm1/Pwp1/Cnbp/Xpot/Rsl1d1/Set/Etf1/Nop56/Mrps18b/Rad23b/Ddx21/Prdx3/Iars/Nop16/Ifrd1/Rack1/Tufm/Ctps/Rps2/Myc/Nolc1/Syncrip/H2az1/Cct2/Fbl/Txnl4a/Rps5/Ube2e1/Cct4/Rrp9/Phb2/Clns1a/Hspd1/Tcp1/Orc2/Dhx15/Hspe1/Abce1/Prpf31/Serbp1/Hddc2/Rpl6/Rps6/Canx/Exosc7/Nme1/Txnl4a/Eef1b2/Cox5a/Psma2/Mcm6/Hnrnpa3/Mcm5
HALLMARK_MTORC1_SIGNALING	0.00131355	2.18233665	Slc7a11/Niban1/Psph/Cth/Trib3/Hspa9/Nupr1/Phgdh/Mthfd2/Slc1a4/Add3/Psat1/Got1/Shmt2/Slc7a5/Eprs/Ddit3/Wars1/Ddit4/Asns
HALLMARK_IL6_JAK_STAT3_SIGNALING	0.00304743	2.2943616	Ccr1/Hmox1/Jun/Il12rb1/Il3ra/Cd14/Crlf2/Csf2/Itgb3/Il10rb/Csf1
HALLMARK_INTERFERON_ALPHA_RESPONSE	0.00924747	2.0156278	Oas1g/Lgals3bp/Psmb8/Rtp4/Wars1/C1s1/Isg15/Txnip/Csf1/Plscr1/Oasl1/Usp18/B2m/Il4ra/Tap1
HALLMARK_P53_PATHWAY	0.01184031	1.90903584	Slc7a11/Hmox1/Trib3/Nupr1/Atf3/F2r/Jun/Klk8/Slc3a2/Nol8/Ptpre/Ddit3/Coq8a/Ddit4/Txnip/Irag2/Retsat/Notch1
HALLMARK_APOPTOSIS	0.02520079	1.92749049	Hmox1/Cth/Atf3/F2r/Jun/Sptan1/Ddit3/Cd14/Txnip/Retsat/Lgals3/H1f0/Etf1/Pmaip1/Psen2/Gna15/Sqstm1/Tap1/Gch1/Pdgfrb
KEGG_AMINOACYL_TRNA_BIOSYNTHESIS	0.00071611	2.58673069	Rars/Lars/Eprs/Sars/Farsb/Wars1/Nars/Ears2/Yars/Gars/Tars/Dars/Cars/Vars/Iars/Rars2/Aars/Sars2
KEGG_RIBOSOME	0.00235535	2.46235848	Rpl22l1/Rpl12/Rpl10a/Rpsa/Rps11/Rsl24d1/Rps13/Rps2/Rps28/Rps5/Rps16/Rpl3/Rps12/Rpl35/Rpl17/Rps23/Rpl10/Rpl5/Rpl37/Rpl4/Rps26/Rpl32/Rpl8/Rpl6/Rps6/Rpl21/Rps17/Rps18/Rpl29/Rps25
KEGG_SYSTEMIC_LUPUS_ERYTHEMATOSUS	0.00779759	2.32774912	Fcgr1/H4c14/H4c12/H3f3b/Fcgr2b/H3c7/H3c1/C1s1/H3c8/H4c11/H4c16/H4c6/H4c2/H3c4/H4c1/H4c4/H3c11/H4c8
KEGG_ENDOCYTOSIS	0.01300934	-2.1588152	Epn1/Vps37c/Igf1r/Pdcd6ip/Hspa8/Iqsec2/Stambp/Acap3/Ehd1/Cltc/Sh3kbp1/Adrb2/Asap1/Asap2/Arrb1/Iqsec1/Cblb/Ldlr/Met/Psd/Tfrc/Ret
KEGG_ARGININE_AND_PROLINE_METABOLISM	0.01923501	2.19685442	Pycr1/Aldh18a1/Got1/Aldh1b1/Srm/Acy1/Prodh
KEGG_GLYCINE_SERINE_AND_THREONINE_METABOLISM	0.02877373	2.1253592	Psph/Cth/Phgdh/Psat1/Shmt2
KEGG_LEISHMANIA_INFECTION	0.03020277	2.03887797	Fcgr1/Tlr4/Jun/Ncf1/Fcgr2b/Cyba
KEGG_NEUROACTIVE_LIGAND_RECEPTOR_INTERACTION	0.03092325	2.06653084	P2rx3/P2ry2/F2r/Grik4/P2rx7/Cnr2/P2ry1/Adora2b/P2rx4
REACTOME_RRNA_PROCESSING	4.2714E-07	2.9940564	Rpp40/Wdr43/Rpl22l1/Gnl3/Wdr75/Nop58/Tsr1/Exosc1/Thumpd1/Utp14a/Rpl12/Nop14/Gar1/Mphosph10/Nip7/Wdr12/Rpl10a/Ltv1/Wdr36/Nop56/Mrm3/Ddx21/Nop2/Utp18/Rpp38/Rpsa/Rps11/Nol9/Pes1/Nob1/Dimt1/Exosc5/Rps13/Pwp2/Rps2/Utp4/Rps28/Fbl/Rps5/Rps16/Rpl3/Rps12/Wdr46/Rrp9/Rcl1/Rpp14/Rpl35/Rpl17/Ftsj3/Exosc4/Utp25/Rps23/Elac2/Heatr1/Rpl10/Hsd17b10/Utp11/Rpl5/Rpl37/Rpl4/Rps26/Rpl32/Rpl8/Rpl6/Nat10/Rps6/Rpl21/Rps17/Rps18/Exosc7/Noc4l/Rpl29/Rps25/Wdr18/Snu13/Tfb1m/Exosc2/Exosc9/Utp15
REACTOME_TRANSLATION	8.273E-06	2.74643886	Eif4ebp1/Rars/Lars/Eprs/Sars/Rpl22l1/Farsb/Wars1/Nars/Ears2/Yars/Gars/Eif3c/Rpl12/Tars/Eef1e1/Rpl10a/Oxa1l/Etf1/Mrrf/Mrps18b/Dars/Cars/Vars/Iars/Rpsa/Rps11/Rars2/Tufm/Aars/Eif2b3/Rps13/Eif3k/Sars2/Mrpl15/Rps2/Mrpl52/Rps28/Rps5/Rpn2/Rps16/Rpl3/Rps12/Mrpl2/Rpl35/Rpl17/Mrpl19/Mars1/N6amt1/Eef1a1/Rps23/Mrpl24/Rpl10/Eif2b1/Eef2/Mrpl38/Rpl5/Rpl37/Srprb/Mrps35/Iars2/Rpl4/Rps26/Rpl32/Eif3l/Ptcd3/Rpl8/Rpl6/Rps6/Rpl21/Rps17/Rps18/Aimp1/Rpl29/Rps25/Farsa/Eif3e
REACTOME_CYTOSOLIC_TRNA_AMINOACYLATION	8.3601E-05	2.78753142	Rars/Lars/Eprs/Sars/Farsb/Wars1/Nars/Yars/Gars/Tars/Eef1e1/Dars/Cars/Vars/Iars
REACTOME_TRNA_AMINOACYLATION	0.00018543	2.69306712	Rars/Lars/Eprs/Sars/Farsb/Wars1/Nars/Ears2/Yars/Gars/Tars/Eef1e1/Dars/Cars/Vars/Iars/Rars2/Aars/Sars2
REACTOME_RESPONSE_OF_EIF2AK4_GCN2_TO_AMINO_ACID_DEFICIENCY	0.00018543	2.64018011	Trib3/Atf3/Cebpg/Cebpb/Ddit3/Rpl22l1/Asns/Atf4/Rpl12/Impact/Rpl10a/Rpsa/Rps11/Rps13/Rps2/Rps28/Rps5/Rps16/Rpl3/Rps12/Rpl35/Rpl17/Rps23/Rpl10/Rpl5/Rpl37/Rpl4/Rps26/Rpl32/Rpl8/Rpl6/Rps6/Rpl21/Rps17/Rps18/Rpl29/Rps25
REACTOME_METABOLISM_OF_RNA	0.00018543	2.2129275	Psmb8/Rpp40/Mettl1/Eprs/Wdr43/Rpl22l1/Gnl3/Wdr75/Nop58/Tsr1/Exosc1/Thumpd1/Qtrt2/Utp14a/Mettl3/Trmt61a/Rpl12/Nop14/Gar1/Prmt5/Pus1/Mphosph10/Nip7/Xpot/Wdr12/Rpl10a/Ltv1/Set/Etf1/Wdr36/Nop56/Prpf19/Apobec1/Nup43/Mrm3/Ddx21/Nop2/Gtf2f2/Nsun2/Utp18/Rpp38/Rpsa/Rps11/Qtrt1/Nol9/Pes1/Snrpb/Nob1/Dimt1/Exosc5/Rps13/Rnmt/Eif4a3/Pwp2/Nt5c3b/Rps2/Polr2h/Cpsf3/Utp4/Rps28/Trmt1/Fbl/Ctu2/Txnl4a/Rps5/Zfp473/Rps16/Dcps/Rpl3/Rps12/Wdr46/Rrp9/Rcl1/Rpp14/Rpl35/Rpl17/Clns1a/Ftsj3/Exosc4/Utp25/Smg5/Rps23/Elac2/Hnrnpf/Heatr1/Dhx15/Psmb5/Psme1/Rpl10/Trmt10a/Hsd17b10/Trp53rka/Dnajc8/Utp11/Rpl5/Nupl2/Rpl37/Trdmt1/Prpf31/Psmd9/Rpl4/Rps26/Gtf2h1/Pus3/Rpl32/Tsen34/Rpl8/Rpl6/Nat10/Sec13/Rps6/Rpl21/Rps17/Puf60/Prkca/Rps18/Exosc7/Noc4l/Rpl29/Rps25/Pus7/Hbs1l/Wdr18/Snu13/Parn/Lsm4/Lsm10/Adat2/Tnpo1/Tfb1m/Exosc2/Txnl4a/Exosc9/Utp15/Urm1/Ptbp1/Isy1
REACTOME_METABOLISM_OF_AMINO_ACIDS_AND_DERIVATIVES	0.0002872	2.39565278	Pycr1/Psph/Cth/Psmb8/Phgdh/Rars/Gpt2/Slc3a2/Aldh18a1/Psat1/Got1/Lars/Slc7a5/Eprs/Sars/Rpl22l1/Srm/Asns/Bckdha/Rpl12/Prodh/Mri1/Hoga1/Eef1e1/Kyat1/Rpl10a/Mtap/Dars/Asrgl1/Iars/Aspa/Lipt2/Rpsa/Lipt1/Rps11/Rps13/Rps2/Enoph1/Ppm1k/Nags/Rps28/Rps5/Rps16/Rpl3/Rps12/Rpl35/Rpl17/Slc25a13/Shmt1/Mars1/Rps23/Psmb5/Psme1/Rpl10/Hsd17b10/Adi1/Sepsecs/Rpl5/Rpl37/Psmd9/Serinc3/Dld/Rpl4/Rps26/Rpl32/Rpl8/Rpl6/Rps6/Rpl21/Rps17/Dbt/Rps18/Echs1/Aimp1/Rpl29/Rps25
REACTOME_HDMS_DEMETHYLATE_HISTONES	0.00042686	2.60068892	H4c14/H4c12/H3c7/H3c1/H3c8/H4c11/H4c16/H4c6/H4c2/H3c4/H4c1/H4c4/H3c11/H4c8
REACTOME_SELENOAMINO_ACID_METABOLISM	0.00075082	2.46752658	Cth/Rars/Lars/Eprs/Sars/Rpl22l1/Rpl12/Eef1e1/Rpl10a/Dars/Iars/Rpsa/Rps11/Rps13/Rps2/Rps28/Rps5/Rps16/Rpl3/Rps12/Rpl35/Rpl17/Mars1/Rps23/Rpl10/Sepsecs/Rpl5/Rpl37/Rpl4/Rps26/Rpl32/Rpl8/Rpl6/Rps6/Rpl21/Rps17/Rps18/Aimp1/Rpl29/Rps25
REACTOME_CELLULAR_RESPONSE_TO_STARVATION	0.00090086	2.43762947	Trib3/Atf3/Cebpg/Sesn2/Cebpb/Ddit3/Rpl22l1/Asns/Atf4/Itfg2/Rpl12/Impact/Rpl10a/Rpsa/Rps11/Flcn/Rps13/Rps2/Rps28/Rps5/Rps16/Rpl3/Rps12/Rpl35/Rpl17/Rps23/Atp6v0e/Rpl10/Rpl5/Rpl37/Rpl4/Fnip1/Rps26/Rpl32/Rpl8/Rpl6/Sec13/Rps6/Rpl21/Rps17/Rps18/Rpl29/Rps25/Atp6v0b
REACTOME_RMTS_METHYLATE_HISTONE_ARGININES	0.00166174	2.455599	H4c14/H4c12/H3c7/H3c1/H3c8/H4c11/H4c16/Prmt5/H4c6/H4c2/Prmt3/Prmt7/H3c4/H4c1/H4c4/H3c11/H4c8/Prmt1/H3c10/H2ac20/Rps2
REACTOME_TRANSCRIPTIONAL_REGULATION_OF_GRANULOPOIESIS	0.00166174	2.41296641	Il6ra/Myb/H4c14/H4c12/H3f3b/Cbfb/Cebpb/H3c7/H3c1/Spi1/H3c8/H4c11/H4c16/H4c6/H4c2/H3c4/H4c1/H4c4/H3c11/H4c8
REACTOME_CYTOKINE_SIGNALING_IN_IMMUNE_SYSTEM	0.00166174	2.05079559	Ccr1/Fcgr1/Oas1g/Havcr2/Il6ra/Hmox1/Tnfrsf13b/Hspa9/Psmb8/Mt2/Gsto1/Jun/Trim34a/Lck/Fgf2/Il12rb1/Il1rl1/Il3ra/Fyn/H3c7/H3c1/Trim35/Crlf2/Dusp6/Csf2/Isg15/Il10rb/H3c8/Car1/Trim46/Csf1r/Lcp1/Rigi
REACTOME_EUKARYOTIC_TRANSLATION_ELONGATION	0.00173899	2.40011112	Rpl22l1/Rpl12/Rpl10a/Rpsa/Rps11/Rps13/Rps2/Rps28/Rps5/Rps16/Rpl3/Rps12/Rpl35/Rpl17/Eef1a1/Rps23/Rpl10/Eef2/Rpl5/Rpl37/Rpl4/Rps26/Rpl32/Rpl8/Rpl6/Rps6/Rpl21/Rps17/Rps18/Rpl29/Rps25
REACTOME_NONSENSE_MEDIATED_DECAY_NMD	0.00337302	2.25586605	Rpl22l1/Rpl12/Rpl10a/Etf1/Rpsa/Rps11/Rps13/Eif4a3/Rps2/Rps28/Rps5/Rps16/Rpl3/Rps12/Rpl35/Rpl17/Smg5/Rps23/Rpl10/Rpl5/Rpl37/Rpl4/Rps26/Rpl32/Rpl8/Rpl6/Rps6/Rpl21/Rps17/Rps18/Rpl29/Rps25
REACTOME_CILIUM_ASSEMBLY	0.00388842	-2.1737441	Nde1/Cep164/Sclt1/Gbf1/Clasp1/Ift140/Hdac6/Dync2i1/Dynll1/Cep135/Tubg1/Kif3c/Cep152/Cep250/Kif3a/Tuba1c/Ninl/Ift22/Lztfl1/Prkar2b/Tuba1b/Ttbk2/Cep89/Bbs2/Nphp1/Cep290/Ywhae/Bbs1/Prkaca/Cetn2/B9d1/Ttc26/Tubb1/Rpgrip1l/Tubb3/Asap1/C2cd3/Tctn1/Tubb4b/Tubb2a/Kif24/Ift122/Wdr35/Tuba4a
REACTOME_ANCHORING_OF_THE_BASAL_BODY_TO_THE_PLASMA_MEMBRANE	0.00407005	-2.2937532	Alms1/Nde1/Cep164/Sclt1/Clasp1/Dynll1/Cep135/Tubg1/Cep152/Cep250/Ninl/Prkar2b/Ttbk2/Cep89/Nphp1/Cep290/Ywhae/Prkaca/Cetn2/B9d1/Rpgrip1l/C2cd3/Tctn1/Tubb4b/Kif24/Tuba4a
REACTOME_EUKARYOTIC_TRANSLATION_INITIATION	0.00480326	2.27378069	Eif4ebp1/Rpl22l1/Eif3c/Rpl12/Rpl10a/Rpsa/Rps11/Eif2b3/Rps13/Eif3k/Rps2/Rps28/Rps5/Rps16/Rpl3/Rps12/Rpl35/Rpl17/Rps23/Rpl10/Eif2b1/Rpl5/Rpl37/Rpl4/Rps26/Rpl32/Eif3l/Rpl8/Rpl6/Rps6/Rpl21/Rps17/Rps18/Rpl29/Rps25/Eif3e
REACTOME_SRP_DEPENDENT_COTRANSLATIONAL_PROTEIN_TARGETING_TO_MEMBRANE	0.00547195	2.28240405	Rpl22l1/Rpl12/Rpl10a/Rpsa/Rps11/Rps13/Rps2/Rps28/Rps5/Rpn2/Rps16/Rpl3/Rps12/Rpl35/Rpl17/Rps23/Rpl10/Rpl5/Rpl37/Srprb/Rpl4/Rps26/Rpl32/Rpl8/Rpl6/Rps6/Rpl21/Rps17/Rps18/Rpl29/Rps25
REACTOME_ATF4_ACTIVATES_GENES_IN_RESPONSE_TO_ENDOPLASMIC_RETICULUM_STRESS	0.0061307	2.33088594	Herpud1/Atf3/Cebpg/Cebpb/Ddit3/Asns/Exosc1/Atf4
REACTOME_SIGNALING_BY_INTERLEUKINS	0.0061307	2.04486412	Ccr1/Havcr2/Il6ra/Hmox1/Hspa9/Psmb8/Gsto1/Jun/Lck/Fgf2/Il12rb1/Il1rl1/Il3ra/Fyn/H3c7/H3c1/Crlf2/Dusp6/Csf2/Il10rb/H3c8/Car1/Csf1r/Lcp1
REACTOME_PERK_REGULATES_GENE_EXPRESSION	0.0064793	2.27140357	Herpud1/Atf3/Cebpg/Cebpb/Ddit3/Asns/Exosc1/Atf4
REACTOME_RUNX1_REGULATES_TRANSCRIPTION_OF_GENES_INVOLVED_IN_DIFFERENTIATION	0.0064793	2.2111988	Psmb8/Myb/H4c14/H4c12/H3f3b/Cbfb/H3c7/H3c1/Spi1/H3c8/H4c11/H4c16/H4c6/H4c2/H3c4/H4c1/H4c4/H3c11/H4c8
REACTOME_SENESCENCE_ASSOCIATED_SECRETORY_PHENOTYPE_SASP	0.00665908	2.19246459	Jun/H4c14/H4c12/H3f3b/Cebpb/H3c7/H3c1/H3c8/H4c11/H4c16/H4c6/H4c2/Anapc16/H3c4/H4c1/H4c4/H3c11/H4c8
REACTOME_CONDENSATION_OF_PROPHASE_CHROMOSOMES	0.00728865	2.25689083	H4c14/H4c12/H3f3b/H3c7/H3c1/H3c8/H4c11/H4c16/H4c6/H4c2/Set/H3c4/H4c1/H4c4/H3c11/H4c8
REACTOME_ACTIVATED_PKN1_STIMULATES_TRANSCRIPTION_OF_AR_ANDROGEN_RECEPTOR_	0.00779374	2.21816312	H4c14/H4c12/H3f3b/H3c7/H3c1/H3c8/H4c11/H4c16/H4c6/H4c2/H3c4/H4c1/H4c4/H3c11/H4c8
REACTOME_MEIOTIC_RECOMBINATION	0.00848703	2.18513715	H4c14/H4c12/H3f3b/H3c7/H3c1/H3c8/H4c11/H4c16/H4c6/H4c2/Top3a/H3c4/H4c1/H4c4/H3c11/H4c8
REACTOME_RRNA_MODIFICATION_IN_THE_NUCLEUS_AND_CYTOSOL	0.0137793	2.18210639	Wdr43/Wdr75/Nop58/Thumpd1/Utp14a/Nop14/Gar1/Mphosph10/Wdr36/Nop56/Nop2/Utp18/Dimt1/Pwp2/Rps2/Utp4/Fbl/Wdr46/Rrp9/Rcl1/Utp25/Heatr1/Utp11/Nat10/Rps6/Noc4l/Snu13/Utp15
REACTOME_METABOLISM_OF_WATER_SOLUBLE_VITAMINS_AND_COFACTORS	0.0137793	2.14688518	Aldh1l2/Mthfd2/Gsto1/Ptgis/Shmt2/Pnpo/Fpgs/Mthfd1l/Qprt/Enpp1/Pccb/Gsto2/Pank1
REACTOME_B_WICH_COMPLEX_POSITIVELY_REGULATES_RRNA_EXPRESSION	0.0137793	2.10810087	H4c14/H4c12/H3f3b/H3c7/H3c1/H3c8/H4c11/H4c16/H4c6/H4c2/H3c4/H4c1/H4c4/Ddx21/H3c11/H4c8/Polr1f/Polr1c/H3c10/H2ac20/Polr2h/Polr1b/H2az1
REACTOME_RHO_GTPASES_ACTIVATE_PKNS	0.0137793	2.1000994	H4c14/H4c12/H3f3b/Pkn3/H3c7/H3c1/H3c8/H4c11/H4c16/H4c6/H4c2/H3c4/H4c1/H4c4/H3c11/H4c8
REACTOME_MEIOSIS	0.0137793	2.06001857	H4c14/H4c12/H3f3b/H3c7/H3c1/H3c8/H4c11/H4c16/H4c6/H4c2/Top3a/H3c4/H4c1/H4c4/H3c11/H4c8/Sun2/H3c10/H2ac20/Stag2/H2az1
REACTOME_RNA_POLYMERASE_I_PROMOTER_ESCAPE	0.01523667	2.08878006	H4c14/H4c12/H3f3b/H3c7/H3c1/H3c8/H4c11/H4c16/H4c6/H4c2/H3c4/H4c1/H4c4/H3c11/H4c8/Polr1f/Polr1c/H3c10/H2ac20/Polr2h/Polr1b/H2az1
REACTOME_PRC2_METHYLATES_HISTONES_AND_DNA	0.0173154	2.09461304	H4c14/H4c12/H3f3b/H3c7/H3c1/H3c8/H4c11/H4c16/H4c6/H4c2/H3c4/H4c1/H4c4/H3c11/H4c8
REACTOME_REGULATION_OF_EXPRESSION_OF_SLITS_AND_ROBOS	0.0173154	2.05002936	Psmb8/Rpl22l1/Rpl12/Rpl10a/Etf1/Rpsa/Rps11/Rps13/Eif4a3/Rps2/Rps28/Rps5/Rps16/Rpl3/Rps12/Rpl35/Rpl17/Rps23/Psmb5/Psme1/Rpl10/Rpl5/Rpl37/Psmd9/Rpl4/Rps26/Rpl32/Rpl8/Rpl6/Rps6/Rpl21/Rps17/Rps18/Rpl29/Rps25
REACTOME_POSITIVE_EPIGENETIC_REGULATION_OF_RRNA_EXPRESSION	0.0173154	2.04685774	H4c14/H4c12/H3f3b/H3c7/H3c1/H3c8/H4c11/H4c16/H4c6/H4c2/H3c4/H4c1/H4c4/Ddx21/H3c11/H4c8/Polr1f/Polr1c/H3c10/H2ac20/Chd3/Polr2h/Polr1b/H2az1
REACTOME_SIRT1_NEGATIVELY_REGULATES_RRNA_EXPRESSION	0.01826248	2.12462046	H4c14/H4c12/H3f3b/H3c7/H3c1/H3c8/H4c11/H4c16/H4c6/H4c2/H3c4/H4c1/H4c4/H3c11/H4c8
REACTOME_MITOCHONDRIAL_PROTEIN_IMPORT	0.01826248	2.10618278	Hspa9/Chchd4/Chchd5/Timm8a1/Timm10/Gfer/Timm9/Tomm20/Chchd10/Chchd3/Hscb/Slc25a13/Hspd1/Coq2/Timm50/Timm44/Fxn/Atp5b
REACTOME_REPRODUCTION	0.01826248	1.98216524	H4c14/H4c12/H3f3b/H3c7/H3c1/H3c8/H4c11/H4c16/H4c6/H4c2/Top3a/H3c4/H4c1/H4c4/H3c11/H4c8/Sun2/H3c10/H2ac20/Catsperd/Stag2/H2az1
REACTOME_INFLUENZA_INFECTION	0.01826248	1.9654852	Rpl22l1/Isg15/Rpl12/Rpl10a/Nup43/Gtf2f2/Rpsa/Rps11/Rps13/Rps2/Polr2h/Rps28/Rps5/Rps16/Rpl3/Rps12/Rpl35/Ipo5/Rpl17/Rps23/Rpl10/Rpl5/Nupl2/Rpl37/Rpl4/Rps26/Rpl32/Rpl8/Rpl6/Sec13/Rps6/Canx/Rpl21/Rps17/Rps18/Rpl29/Rps25
REACTOME_ESTROGEN_DEPENDENT_GENE_EXPRESSION	0.01826248	1.94986435	Myb/Jun/H4c14/H4c12/H3f3b/Cbfb/H3c7/H3c1/H3c8/H4c11/H4c16/H4c6/H4c2/H3c4/H4c1/H4c4/Gtf2f2/H3c11/H4c8/Prmt1/H3c10/H2ac20/Polr2h/Myc/Stag2
REACTOME_ASSEMBLY_OF_THE_ORC_COMPLEX_AT_THE_ORIGIN_OF_REPLICATION	0.01836163	2.1162498	H4c14/H4c12/H3f3b/H3c7/H3c1/H3c8/H4c11/H4c16/H4c6/H4c2/H3c4/H4c1/H4c4/H3c11/H4c8
REACTOME_ERCC6_CSB_AND_EHMT2_G9A_POSITIVELY_REGULATE_RRNA_EXPRESSION	0.01836163	2.06043519	H4c14/H4c12/H3f3b/H3c7/H3c1/H3c8/H4c11/H4c16/H4c6/H4c2/H3c4/H4c1/H4c4/H3c11/H4c8/H3c10/H2ac20/Chd3
REACTOME_ANTIGEN_PROCESSING_CROSS_PRESENTATION	0.01967071	2.09014103	Fcgr1/Tlr4/Psmb8/Ncf1/Cd14/Tlr6/Cyba
REACTOME_SIGNALING_BY_HEDGEHOG	0.01967071	-1.9980391	Sem1/Kif3a/Iqce/Tuba1c/Tulp3/Syvn1/Prkar2b/Tuba1b/Ptch1/Spopl/Ulk3/Prkaca/Tubb1/Rpgrip1l/Fuz/Tubb3/Tubb4b/Tubb2a/Ift122/Arrb1/Wdr35/Tuba4a
REACTOME_ESR_MEDIATED_SIGNALING	0.01967071	1.92228148	Myb/Jun/H4c14/H4c12/Btc/H3f3b/Cbfb/Gng2/H3c7/H3c1/H3c8/H4c11/H4c16/Gng5/H4c6/H4c2/H3c4/H4c1/H4c4/Srf/Src/Gtf2f2/H3c11/H4c8
REACTOME_RECYCLING_PATHWAY_OF_L1	0.02054875	-1.9809928	Msn/Cltc/Tubb1/Tubb3/Dpysl2/Tubb4b/Rps6ka6/Tubb2a/Tuba4a
REACTOME_AMYLOID_FIBER_FORMATION	0.02062115	1.98672011	H4c14/Lyz2/H4c12/H3f3b/H3c7/H3c1/H3c8/Tspan5/H4c11/H4c16/H4c6/H4c2/Prkn/H3c4/H4c1/H4c4/H3c11/H4c8
REACTOME_ORGANELLE_BIOGENESIS_AND_MAINTENANCE	0.02150185	-1.8172597	Alms1/Alas1/Nde1/Cep164/Sclt1/Gbf1/Clasp1/Ift140/Atp5j/Hdac6/Dync2i1/Dynll1/Cep135/Polg2/Tubg1/Kif3c/Cep152/Cep250/Kif3a/Ncor1/Tuba1c/Ninl/Usp46/Ift22/Lztfl1/Prkar2b/Tuba1b/Ttbk2/Micos10/Cep89/Ncoa6/Bbs2/Crebbp/Nphp1/Cep290/Ywhae/Bbs1/Chd9/Hcfc1/Prkaca/Cetn2/B9d1/Ttc26/Tubb1/Rpgrip1l/Tubb3/Asap1/C2cd3/Tctn1/Tubb4b/Tubb2a/Kif24/Ift122/Wdr35/Tuba4a
REACTOME_METABOLISM_OF_VITAMINS_AND_COFACTORS	0.02205136	1.9941867	Aldh1l2/Mthfd2/Gsto1/Ptgis/Shmt2/Pnpo/Fpgs/Mthfd1l/Vkorc1/Qprt/Retsat/Enpp1/Pccb/Gsto2/Pank1
REACTOME_CELLULAR_RESPONSES_TO_STIMULI	0.02657795	1.66273981	Chac1/Tlr4/Hmox1/Trib3/Herpud1/Hspa9/Psmb8/Atf3/Mt2/Jun/H4c14/H1f1/H4c12/Mt1/H3f3b/Ncf1/Cebpg/Cited2/Sesn2/Cebpb/Gml2/Ddit3/Rpl22l1/H3c7/H3c1/Asns/Aqp8/Exosc1/Atf4/Cyba/Tubb2b/Txnip/Id1/H3c8/H4c11/Sco2/H4c16/Itfg2/Ero1a/H1f0/Dnajb9/Rpl12/H4c6/H4c2/Impact/Rpl10a/Anapc16/H3c4/H4c1/H4c4/Nup43/Prdx3/H1f3/H13/Rnf2
REACTOME_LEISHMANIA_INFECTION	0.02711834	1.94648017	Fcgr1/Hmox1/Jun/Adm2/Gng2/Fcgr2b/Fyn/P2rx7/Cyba/Txnip/Gng5/Adora2b/P2rx4/Src/Itpr1/Plcg2
REACTOME_MITOTIC_PROPHASE	0.02711834	1.88617062	Nek6/H4c14/H4c12/H3f3b/H3c7/H3c1/H3c8/H4c11/H4c16/H4c6/H4c2/Set/H3c4/H4c1/H4c4/Nup43/H3c11/H4c8
REACTOME_REGULATION_OF_PLK1_ACTIVITY_AT_G2_M_TRANSITION	0.02737661	-1.9571819	Alms1/Nde1/Cep164/Clasp1/Dynll1/Cep135/Tubg1/Ccnb2/Cep152/Cep250/Ninl/Prkar2b/Cep290/Ywhae/Prkaca/Cetn2/Ppp1r12b/Tubb4b/Tuba4a
REACTOME_DNA_METHYLATION	0.03165384	2.03229431	H4c14/H4c12/H3f3b/H3c7/H3c1/H3c8/H4c11/H4c16/H4c6/H4c2/H3c4/H4c1/H4c4/H3c11/H4c8
REACTOME_RNA_POLYMERASE_I_TRANSCRIPTION	0.03659352	1.9080976	H4c14/H4c12/H3f3b/H3c7/H3c1/H3c8/H4c11/H4c16/H4c6/H4c2/H3c4/H4c1/H4c4/H3c11/H4c8/Polr1f/Polr1c/H3c10/H2ac20/Chd3/Polr2h/Polr1b/H2az1
REACTOME_GPCR_LIGAND_BINDING	0.03687902	2.01502707	Ccr1/P2ry2/F2r/Adm2/Gng2/Cnr2/Prokr1/Gng5/P2ry1/Cxcr4/Adora2b
REACTOME_RECRUITMENT_OF_NUMA_TO_MITOTIC_CENTROSOMES	0.03687902	-1.8808486	Alms1/Nde1/Cep164/Clasp1/Dynll1/Cep135/Tubg1/Cep152/Cep250/Tuba1c/Ninl/Prkar2b/Tuba1b/Cep290/Ywhae/Prkaca/Cetn2/Tubb1/Tubb3/Nme7/Tubb4b/Tubb2a/Tuba4a
REACTOME_GENE_SILENCING_BY_RNA	0.0380604	1.83682475	H4c14/H4c12/H3f3b/H3c7/H3c1/H3c8/Tdrd9/H4c11/H4c16/H4c6/H4c2/H3c4/H4c1/H4c4/Nup43/Xpo5/H3c11/H4c8
REACTOME_MITOTIC_G2_G2_M_PHASES	0.03997258	-1.8172085	Psma2/Alms1/Nde1/Cenpf/Cep164/Clasp1/Dynll1/Cep135/Mybl2/Tubg1/Foxm1/Lin37/Ccnb2/Cep152/Cep250/Ppp2r3d/Sem1/Tuba1c/Ninl/Prkar2b/Tuba1b/Fbxl18/Cep290/Ywhae/Trp53/Prkaca/Cetn2/Ep300/Tubb1/Ppp1r12b/Tubb3/Nme7/Cdc25b/Tubb4b/Tubb2a/Tuba4a
